# Genetic control of seasonal meristem arrest in trees

**DOI:** 10.1073/pnas.2505641122

**Published:** 2025-11-26

**Authors:** Jun Wang, Xiaoli Liao, Zhihao Wu, Shashank Sane, Shaopeng Han, Qihui Chen, Xueping Shi, Xiaokang Dai, Maria Klintenäs, Ove Nilsson, Jihua Ding

**Affiliations:** ^a^Hubei Hongshan Laboratory, Hubei Engineering Technology Research Center for Forestry Information, College of Horticulture and Forestry Sciences, Huazhong Agricultural University, Wuhan 430070, China; ^b^Umeå Plant Science Centre, Department of Forest Genetics and Plant Physiology, Swedish University of Agricultural Sciences, Umeå 901 83, Sweden

**Keywords:** *Populus*, seasonal growth, meristem arrest, *APETALA2*

## Abstract

Perennial plants, such as trees native to temperate and boreal regions, exhibit meristems that undergo annual cycles of activity and rest to synchronize their growth cycles with seasonal changes, ensuring survival under harsh winter conditions. The arrest of shoot meristem growth, known as growth cessation, is a critical initial step for trees to enter dormancy. This process is triggered by a combination of endogenous and exogenous signals, yet the molecular mechanisms and signaling pathways underlying growth cessation remain poorly understood. In this study, we demonstrate that *Populus* orthologs of APETALA2-like transcription factors (AP2Ls), the primary regulators of global proliferative arrest (GPA) in *Arabidopsis*, play a crucial role in the regulation of seasonal growth cessation in hybrid aspen trees. In particular, AP2Ls act as important activators of the expression of *FLOWERING LOCUS T2* (*FT2*), a key gene for short-day-induced growth cessation. This contrasts with the established role of AP2Ls as repressors of *FT* in annual plants. Yet, the pathway itself is conserved with the pathway regulating GPA in annual plants, a completely different process during the plant life cycle. Our research highlights both the conserved roles and functional diversities of *AP2Ls* in a more general balancing of meristem proliferation and arrest in perennial plants, providing insights into the evolutionary adaptation of growth regulation mechanisms across plant species.

## Significance

Shoot stem cells are precursors of aboveground tissues. A precise regulatory basis is required to control their activity during plant development. This study reveals that APETALA2-like transcription factors in hybrid aspen play a central role in regulating annual growth arrest, a critical adaptation for perennial trees in temperate and boreal regions. These findings uncover a conserved yet functionally diversified genetic pathway that balances meristem proliferation and arrest, bridging the regulatory mechanisms of growth cessation in perennial plants and global proliferative arrest in annual plants. This work advances our understanding of the molecular basis of seasonal growth regulation and highlights the evolutionary adaptation of growth control strategies across plant species.

In flowering plants, shoot stem cells are precursors of aboveground tissues. A precise regulatory basis is required to ensure them in the right status during plant development. In monocarpic plants, for example, the end of reproductive phase is determined by a coordinated arrest of all active inflorescence meristems (IM) named global proliferative arrest (GPA) (1). In contrast, shoot meristems in polycarpic perennials cycle between two different states: In one state they maintain a supply of vegetative meristems (VMs) to sustain growth of the plant in future growth cycles. In the other state the VMs undergo growth cessation and transit into dormancy at the end of a season for winter survival, especially in long-lived trees native to boreal and temperate regions (2, 3). The monocarpic plant GPA is a one-way process that triggers a global senescence program and cell death in the IM (1), while the seasonal growth cessation and dormancy in perennial plants is a transient state where the VM goes through a meristematic arrest and enters a “quiescence” or “rest” phase (4). Cell proliferation can resume once the suppression conditions are relieved. In *Arabidopsis*, GPA is regulated by the *APETALA2 (AP2)* and *APETALA2-like* genes of the euAP2 lineage in the shoot apical meristem (SAM) (5). The expression of *AP2-like* genes is negatively regulated by *FRUITFULL* (*FUL*), a MADS-box gene involved in flowering and fruit development (5), and *microRNA172* (*miR172*), a conserved miRNA in plants (6). These genes act as master regulators of SAM activity maintenance by coordinating endogenous hormone pathways and environmental signals (7). However, the molecular base of seasonal VM growth cessation in perennial trees is still poorly understood.

In the model perennial plant *Populus*, the timing of annual growth cessation in the shoot meristem during autumn is primarily governed by short-day (SD) photoperiods (8). FLOWERING LOCUS T2 (FT2), an evolutionary conserved protein in flowering induction, plays a central role in this process (9, 10). *FT2* is expressed in leaves and is induced by long-days (LD). FT2 proteins travel through the phloem from leaves to shoot apices to sustain vegetative growth (11). Conversely, SD photoperiods lead to a rapid decrease in *FT2* expression in leaves thus triggering growth cessation (12). In the shoot apex, the pathway involving *FT2* in maintaining shoot meristem activity has been partially elucidated: The FT2 protein translocates to the shoot apex and forms a complex with FD-LIKE1 (FDL1), leading to the activation of *Like-APETALA1* (*LAP1*) (13). LAP1, in turn, directly induces *AINTEGUMENTA-LIKE 1* (*AIL1*) (14), which regulates the expression of D-type cyclins to facilitate cell-cycle progression. However, the existing studies do not fully account for the significant impact of *FT2* on SAM activity. Trees lacking *FT2* are unable to sustain growth (15), while the effect on growth cessation in *LAP1RNAi* plants is much weaker (13, 16). These findings suggest the presence of yet unidentified genes that could link *FT2* and SAM activity maintenance by integrating the aforementioned pathways. Based on transcriptomic analysis during the GPA process in *Arabidopsis*, it has been proposed that arrested meristems behave as dormant meristems, since they can recover their activity after seed removal (17). The molecular dissection of *AP2* further supports that the process of GPA resembles a dormant meristem (7). Given the crucial roles of the *miR172-AP2* module in the regulation of meristem proliferative arrest in monocarpic plants, one could speculate about its potential involvement in the regulation of seasonal growth cessation in perennial trees, where its functions are not well understood.

Here, we show that *miR172* and its targets, the *Populus* AP2-like(AP2L) transcription factors (TFs), play an essential role in the seasonal growth cessation process in *Populus* trees. The *miR172/AP2L* regulatory module coordinates the regulation of growth cessation by serving as a central regulator of the key gene *FT2* in leaves and core cell cycle genes involved in the replication machinery and cell expansion regulators in shoots. This finding sheds light on the intricate network of genetic factors involved in orchestrating the transition to growth cessation in perennial trees and provides valuable insights into the molecular mechanisms underlying this critical developmental process. Furthermore, the fact that this genetic network is conserved in the regulation of a seemingly completely different developmental process in annual plants; the induction of GPA after flowering and fruit set, suggests a more general role for these genes in controlling the balance between meristem proliferation and arrest.

## Results

### *miR172* Promotes Growth Cessation in *Populus*.

**Fig. 1.**
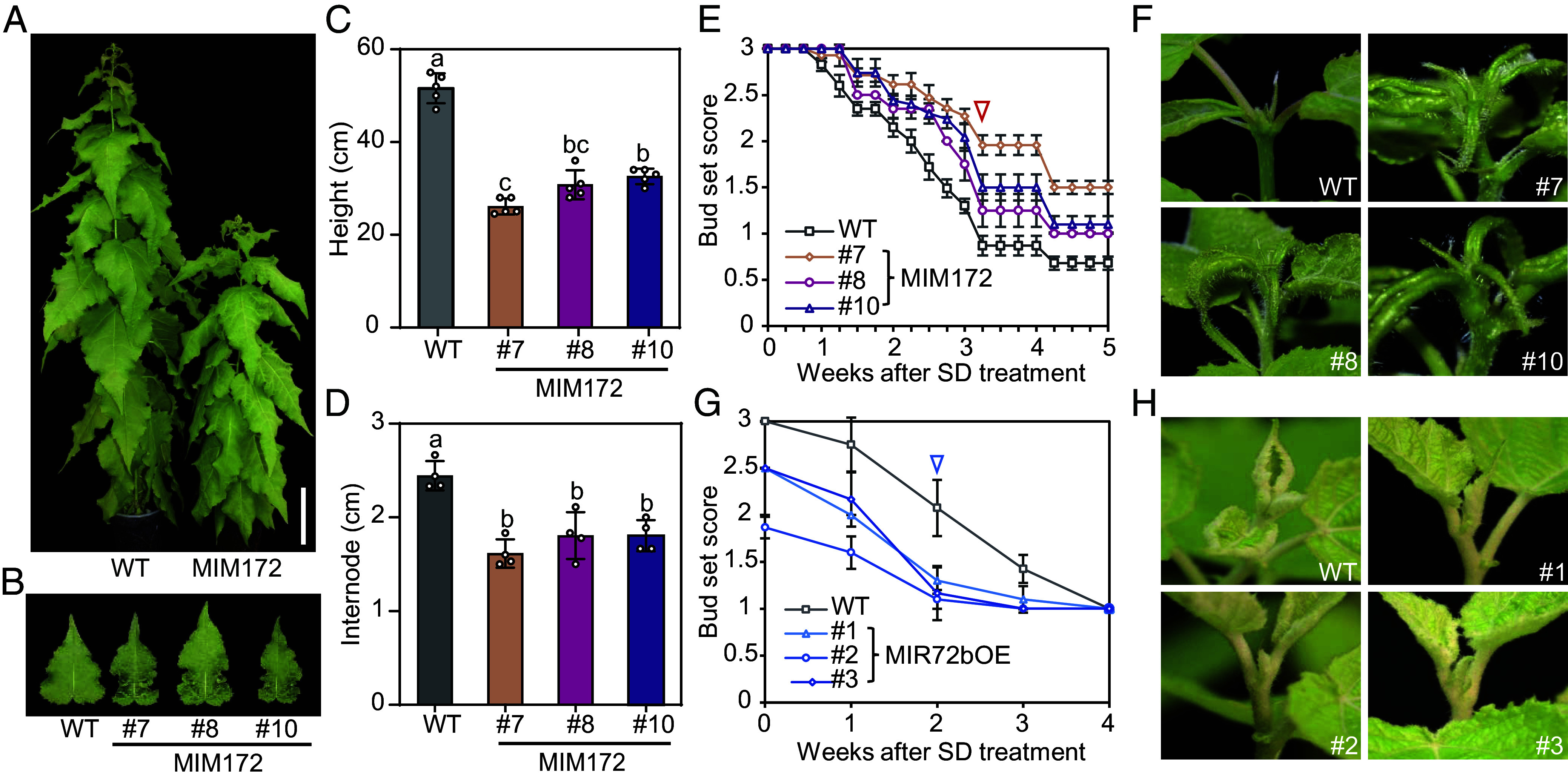
*miR172* promotes growth cessation in *Populus.* (*A*) Overview of WT and MIM172 plants grown in LD^18h^ conditions for 2 mo. (Scale bar, 10 cm.) (*B*–*D*) Phenotypic comparison of WT and MIM172 transgenic lines after 2 mo grown in LD^18h^ conditions, including mature leaf morphology (*B*), plant height (*C*), and internode length (*D*). All measurements were performed on the part of the trees between 20 cm above the soil and 10 cm under the top. The youngest fully expanded leaves (10–12) from the shoot apex were selected to measure the leaf morphology in (*B*). Data shown are mean values from six plants of each line. Error bars ± SE. One-way ANOVA with Tukey’s multiple comparison test was used to determine the significance of differences from WT plants. (*E*) Bud set score of WT and MIM172 plants after transfer from LD^18h^ to SD^8h^ conditions. (*F*) Representative shoot apex morphologies of WT (T89) and MIM172 plants after 3 wk of SD^8h^ treatment. The corresponding score values of shoot apices in (*F*) are marked with a red triangle in (*E*). (*G*) Bud set score of wild type (717) and MIR172bOE plants after transfer from LD^18h^ to SD^8h^ conditions. Data shown in (*E*) and (*G*) are mean values from six plants of each line. Error bars ± SE. (*H*) Representative shoot apex morphologies of WT (717) and MIR172bOE plants after 10 d of SD^8h^ treatment. The corresponding score values of shoot apices in (*H*) are marked with a blue triangle in (*G*).

To see whether the *miR172*/*AP2L* regulatory module might have a role during seasonal growth cessation, we first analyzed the function of *miR172*. In *Populus*, there are nine loci in the *ptc-MIR172* family, named *ptc-MIR172a-i* (*SI Appendix*, Fig. S1*A* and Table S1), with five of them showing detectable and seasonal expression patterns based on the year-round RNA-seq profiling of aspen trees (*SI Appendix*, Fig. S1*B*). Among them, *ptc-MIR172a/b* exhibited predominant expression in leaves during the active growth season, peaking in summer and early autumn when growth cessation is induced. In contrast, *ptc-MIR172c/g/i* were mainly expressed in buds, reaching maximal levels in late autumn and winter. Their temporally complementary profiles suggest that different members of the *miR172* family might play roles during different phenological transitions across the annual growth cycle. To investigate the role of *miR172*, we utilized microRNA target mimicry technology to interfere with *miR172* activity (MIM172) by theoretically targeting all the sequences of the *ptc-MIR172* family (*SI Appendix*, Fig. S2*A*). Three independent MIM172 lines were obtained and potted in the greenhouse (*SI Appendix*, Fig. S2*C*). In LD^18h^ conditions, compared with Wild-type (WT) plants, the MIM172 plants exhibited reduced height, shorter stem internode distances, and curly leaves (Fig. 1 *A*–*D*). We then compared the growth cessation response in MIM172 and WT plants after SD^8h^ treatment (*SI Appendix*, Fig. S3). The MIM172 plants displayed a reduced sensitivity to the daylength shift, with growth cessation occurring about 1 wk later than in WT plants (Fig. 1 *E* and *F*). Additionally, attempts were made to generate *miR172* overexpressors by ectopically expressing the primary transcript of *ptc-MIR172b* (MIR172bOE, *SI Appendix*, Fig. S2*B*). Transgenic plants in the hybrid aspen background (*Populus tremula x tremuloides*, T89) could not be obtained, likely due to the inhibition of regeneration caused by *miR172* during the tissue culture step, as has been reported in other species (18). However, in the hybrid poplar background (*P. tremula x alba*, INRA717-1B4), three independent lines of MIR172bOE plants with modest overexpression levels were successfully generated (*SI Appendix*, Fig. S2*D*). In contrast to MIM172 plants, all these three MIR172bOE lines display earlier growth cessation compared to WT plant (Fig. 1 *G* and *H*). These results strongly suggest that *miR172* functions as a promoter of seasonal growth cessation in *Populus*.

Additionally, we monitored the time of bud break in MIM172 and MIR172bOE plants in controlled growth chambers simulating seasonal changes (*SI Appendix*, Fig. S4*A*). Notably, MIM172 plants exhibited earlier bud break, whereas MIR172bOE plants showed a significant delay compared to WT (*SI Appendix*, Fig. S4 *B*-*E*). These results indicate that *miR172* exerts pleiotropic effects on seasonal growth regulation, aligning with the distinct seasonal expression patterns of *ptc-MIR172* isoforms observed earlier (*SI Appendix*, Fig. S1*B*).

### *AP2-like* Genes Function Redundantly in Growth Cessation Control.

Six AP2-like TFs were found as putative targets of *miR172* in *Populus*, including two *APETALA2-Like* genes (*APETALA2-Like1*, *AP2L1* and *APETALA2-Like2*, *AP2L2*) that are orthologous to *AP2* in *Arabidopsis*, and four *TOE-Like* (*TOEL1-4*) genes that grouped together with *Arabidopsis TOE1*, where *TOEL1/2* and *TOEL3/4* are two pairs of paralogs (*SI Appendix*, Fig. S5 *A* and *B*). These are the only genes likely to be targets of *miR172* in *Populus* trees (*SI Appendix*, Table S1 and Dataset S1). AP2L/TOEL share high amino acid sequence similarity, containing two conserved EAR domains and an AP2 domain (*SI Appendix*, Fig. S5*C*). The predicted target sites were located near the 3′ end of the coding sequence (CDS) and were also conserved at the protein level (*SI Appendix*, Fig. S5*C*). While *TOEL1/2* were elevated in MIM172 lines, the transcript levels of *AP2L1/2* and *TOEL3/4* remained unchanged (*SI Appendix*, Fig. S6). This observation suggests that *miR172* may regulate AP2-like TFs not only through transcript cleavage but also via translational repression, which aligns with previous reports in other species (19–21).

**Fig. 2.**
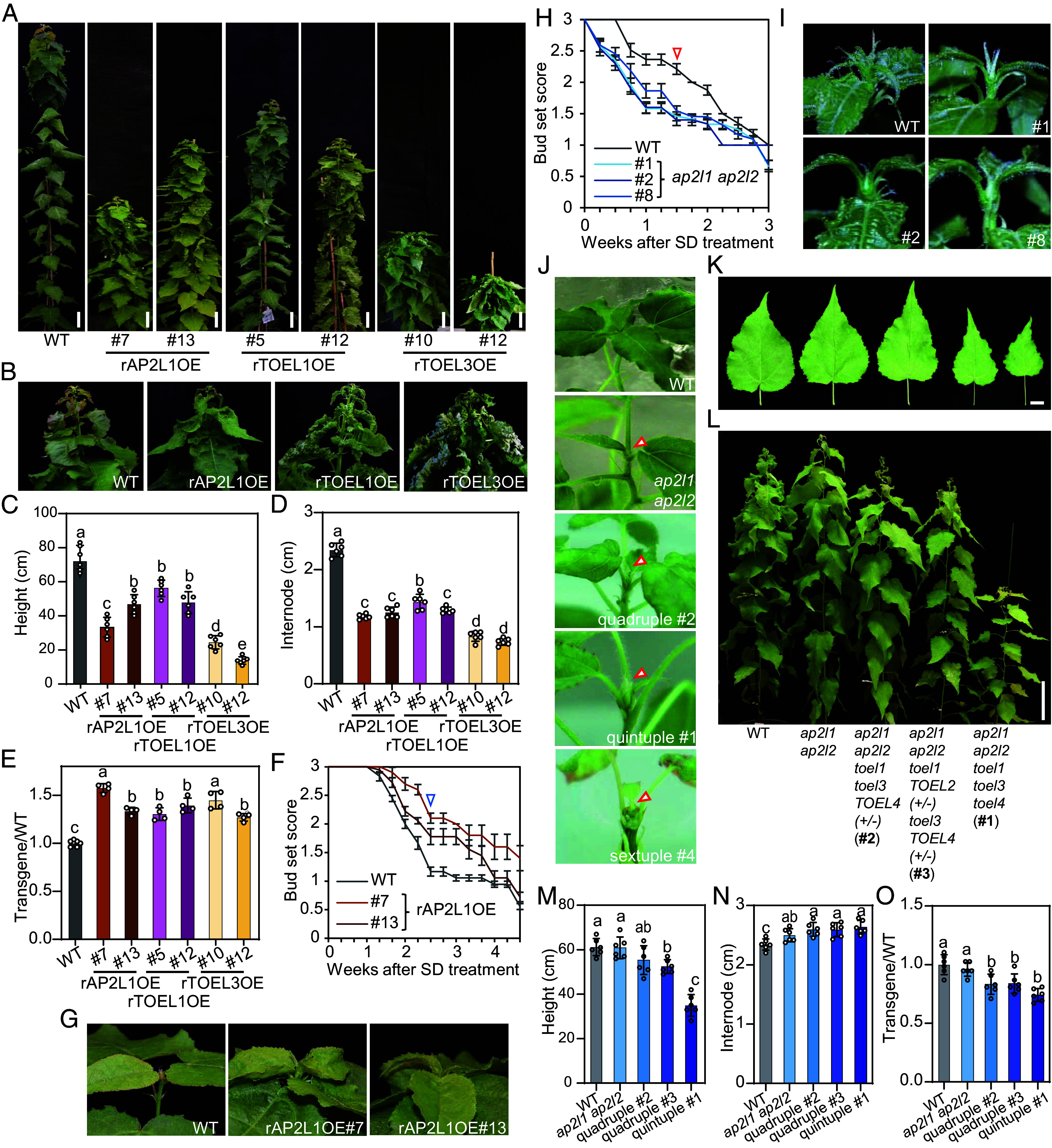
*AP2L/TOEL* function redundantly in growth cessation control. (*A*) Overview of WT and rAP2L1OE, rTOEL1OE, and rTOEL3OE plants grown under LD^18h^ conditions for 3 mo. (Scale bar, 10 cm.) (*B*–*D*) Phenotypic comparison of WT, rAP2L1OE, rTOEL1OE, and rTOEL3OE transgenic plants after 3 mo grown in LD^18h^ conditions, including leaf and shoot apex morphology (*B*), plant height (*C*), and internode length (*D*). (*E*) Relative leaf number in *AP2L/TOEL* overexpressors compared to WT plants after 3 mo of growth in LD^18h^ conditions. Data shown in (*C*–*E*) are mean values from six plants of each line. Error bars ± SE. (*F*) Bud set score of WT and rAP2L1OE plants after transfer from LD^18h^ to SD^8h^ conditions. (*G*) Representative shoot apex morphologies of WT and rAP2L1OE plants after 3 wk of SD^8h^ treatment. (*H*) Bud set score of WT and *ap2l1 ap2l2* plants after transfer from LD^18h^ to SD^8h^ conditions. (*I*) Representative shoot apex morphologies of WT and *ap2l1 ap2l2* plants after 2 wk of SD^8h^ treatment. The corresponding score values of shoot apices in (*G*) and (*H*) are marked with a blue triangle in (*F*) and a red triangle in (*I*), respectively. Data shown in (*F*) and (*H*) are mean values from six to eight plants of each line. Error bars ± SE. (*J*) Shoot apex morphologies of WT, *ap2l1 ap2l2,* and quadruple, quintuple, and sextuple mutants of *AP2L/TOEL* in tissue culture after 8 wk of amplification. Shoot apices are highlighted with red triangles. (*K*) Overview of *AP2L*/*TOEL* mutants after 2 mo of growth in LD^18h^ conditions. The youngest fully expanded leaves (10–12) from the shoot apex were selected to measure the leaf morphology. (Scale bar, 10 cm.) (*L*–*O*) Phenotypic comparison of WT and *AP2L*/*TOEL* mutants after 2 mo grown in LD^18h^ conditions, including leaf morphology (*L*), plant height (*M*), and internode length (*N*). [Scale bar, 2 cm in (*L*).] (*O*) Relative leaf number in *AP2L*/*TOEL* mutants compared to WT plants after 2 mo of growth in LD^18h^ conditions. Data shown in (*C*–*E*, *M*, and *O*) are mean values from six to eight plants of each line. Error bars ± SE. One-way ANOVA with Tukey’ s multiple comparison test was used to determine the significance of differences between WT and transgenics lines.

To further investigate the role of the AP2-like TFs in growth cessation, miRNA-resistant overexpressor lines were generated for *AP2L1, TOEL1,* and *TOEL3* genes, respectively (rAP2L1OE, rTOEL1OE, and rTOEL3OE; *SI Appendix*, Fig. S7 *A*–*D*). These overexpressor plants exhibited similar phenotypes, including short stature (Fig. 2 *A* and *C*), curly leaves (Fig. 2*B*), and shorter internode distances (Fig. 2*D*). Additionally, the plastochron of the overexpressor plants was reduced, leading to an increased formation of leaves (Fig. 2*E*). The rTOEL1OE and rTOEL3OE plants showed stronger effects on leaf morphology compared to rAP2L1OE plants, indicating predominant roles of *TOELs* in leaf development (Fig. 2*B*). Similar to MIM172 plants, AP2L1OE plants displayed a delayed growth cessation (Fig. 2 *F* and *G*). However, rTOEL1OE and rTOEL3OE plants did not show significant effects on growth cessation (*SI Appendix*, Fig. S7*E*). These results indicate that *AP2L/TOEL* genes exhibit both shared and distinct functions, with *AP2L* genes having a stronger effect on the regulation of growth cessation.

We then generated CRISPR knock-out lines of three pairs of *AP2-like* paralogs, respectively. Three independent *ap2l1 ap2l2* lines, one *toel1 toel2* line, one *toel3 toel4* line, and four *toel* single mutant lines were obtained (*SI Appendix*, Fig. S8). Based on the genome editing results, all three *ap2l1ap2l2* lines produced a truncated protein retaining only the AP2-R1 domain (*SI Appendix*, Fig. S8*A*). This represents a loss-of-function form, as previously verified (22, 23) and further supported in this study by the observed floral defects (*SI Appendix*, Fig. S8*B*). Similarly, *toel1 toel2* and *toel3 toel4* plants are protein null mutants, with the entire AP2 domain absent (*SI Appendix*, Fig. S8 *C* and *D*). We also analyzed the transcript levels of all six *AP2-like* genes in these mutants. While expression showed slight alterations, no consistent trends were observed across the different mutant backgrounds (*SI Appendix*, Fig. S9). Unlike *AP2L/TOEL* overexpressors, the growth morphology of these mutants was similar to WT plants under LD^18h^ conditions (*SI Appendix*, Fig. S10 *A*–*C*). The *ap2l1ap2l2* plants displayed a hypersensitivity to the change in daylength and initiated growth cessation about 1 wk earlier compared to WT (Fig. 2 *H* and *I*). However, there was no significant effect on growth cessation in *TOEL* single or double mutants (*SI Appendix*, Fig. S10*D*). To investigate the possible functional redundancy of *AP2L/TOEL* genes, *TOEL1/2* and/or *TOEL3/4* were knocked out in an *ap2l1ap2l2* mutant background to generate quadruple, quintuple, and sextuple mutants (*SI Appendix*, Fig. S11). We found that the *ap2l1 ap2l2 toel1 toel3* quadruple mutant and the *ap2l1 ap2l2 toel1 toel3 toel4* quintuple mutant already stopped growing in tissue culture (Fig. 2*J*, #1 and #2). Even more extreme, the *ap2l1 ap2l2 toel1 toel2 toel3 toel4* sextuple mutant could not survive in the tissue culture medium (Fig. 2*J*, #4). When these mutants were transferred to the greenhouse, the growth effects became more pronounced with increasing mutations, as evidenced by reduced leaf size and growth rate (Fig. 2 *K*–*M*), longer internode distances, and increased plastochron (Fig. 2 *N* and *O*). Based on both the loss-of-function and overexpression phenotypes, *AP2Ls* are likely to be the most relevant *miR172* targets in terms of affecting SD-induced growth cessation and bud set, with *TOEL* genes functioning redundantly in this process. This conclusion is also supported by comparative transcriptomic analysis showing that >60% of differentially expressed genes (DEGs) in *ap2l1ap2l2* shoot apices overlap with those in MIM172 shoot apices (*SI Appendix*, Fig. S12).

### *AP2L/TOEL* Act as a Hub to Maintain Shoot Apical Activity.

**Fig. 3.**
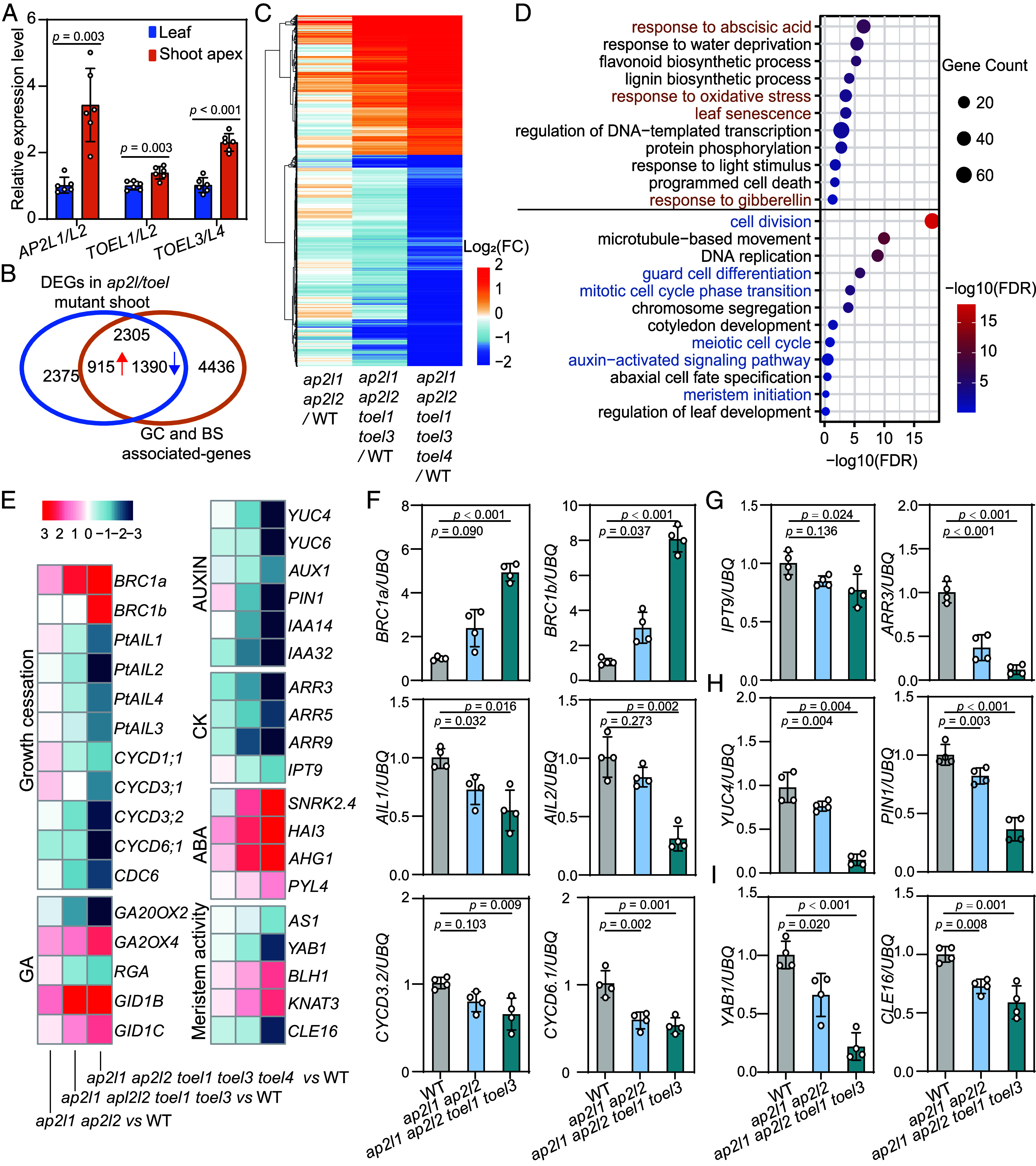
*AP2L/TOEL* act bifunctionally as a hub in keeping shoot apical activity. (*A*) Expression analysis of *AP2L/TOEL* in leaves and shoot apices of plants growing in LD^18h^ conditions. Data shown are mean values from three biological replicates. Error bars ± SE. *U6* and *UBQ* were used as internal reference genes. (*B*) Venn diagram illustrating overlap between DEGs identified in *AP2L/TOEL* mutant lines and genes associate with growth cessation (GC) and bud set (BS). WT*, ap2l1 ap2l2*, *ap2l1 ap2l2 toel1 toel3,* and *ap2l1 ap2l2 toel1 toel3 toel4* shoot apices from LD^18h^ conditions were used for transcriptome dynamic analysis. Two clusters of DEGs that were gradually up- or down-regulated with increasing numbers of gene mutations were selected for analysis. DEGs that identified through comparison of WT shoot apices collected under LD^18h^ conditions versus after 4 wk of SD^8h^ treatment were classified as growth cessation (GC)- and bud set (BS)-associated genes. (*C*) K-means clustering show two groups of common DEGs that are gradually up- or down-regulated with increasing numbers of gene mutations. Log2Foldchange values were used to for heatmap analysis. (*D*) GO analysis of these up and down-regulated DEGs in terms of biological processes (Dataset S2*F*). Biological processes highlighted with brown and blue color were selected for further analysis. (*E*) Heatmap analysis of selected common genes among *ap2l/toel* mutants and WT representing different biological processes. (*F*–*I*) q-PCR analysis of important genes that associated with growth cessation (*BRC1a, BRC1b, AIL1, AIL2, CYCD3;2* and *CYCD6;1*), cytokinin and auxin signals (*IPT9*, *ARR3*, *YUC4,* and *PIN1*), and shoot meristem maintenance and proliferation (*CLE16* and *YAB1*) in WT*, ap2l1 ap2l2,* and *ap2l1 ap2l2 toel1 toel3* shoot apices from LD^18h^ conditions. Data shown are mean values from three biological replicates. Error bars ± SD. One-way ANOVA with the Games–Howell test was used to determine the significance of differences from WT plants.

The *AP2L/TOEL were* expressed in both leaves and shoots, with significantly higher expression levels in the shoot apex (Fig. 3*A*). To gain molecular insights into the mechanisms underlying the roles of *AP2L/TOEL* in regulating shoot apex activity, we performed a transcriptome sequencing (RNA-seq) analysis of *ap2l1 ap2l2*, *ap2l1 ap2l2 toel1 toel3, ap2l1 ap2l2 toel1 toel3 toel4* and WT shoot apices in LD^18h^ conditions. Supervised hierarchical clustering analysis revealed two clusters of genes that showed gradual upregulation (2,002 genes) or downregulation (2,678 genes) as the number of mutants increased (Dataset S2 *A* and *B*). We then overlapped these genes with those DEGs identified in the comparison between LD**^18h^** and SD**^8h^** transcriptomes from WT shoot apices, which are linked to growth cessation and bud set (Dataset S2*C*). As anticipated, nearly 50% (2,305/4,675) of DEGs in *AP2L/TOEL* mutants were identified as growth cessation and bud set associate genes, with 915 upregulated and 1,390 downregulated (Fig. 3 *B* and *C* and Dataset S2 *D* and *E*). Gene ontology (GO) analysis indicated that upregulated genes were mainly involved in stress-related responses such as oxidative stress responses, phytohormone ABA responses, and senescence processes (Dataset S2*F*); while the downregulated genes were mainly related to active growth processes such as cell division, meristem proliferation, and auxin signaling (Fig. 3*D* and Dataset S2*F*). Intriguingly, many genes regulating growth cessation, including *BRANCHED1 (BRC1s)*, *AINTEGUMENTALIKE* (*AILs*), and *D-type cyclins* (*CYCDs*), were either up- or down-regulated in *AP2L/TOEL* mutants (Fig. 3 *E* and *F*). Besides, we observed extensive enrichment of genes associated with phytohormone biosynthesis and signaling, particularly gibberellin (*GA20 OXIDASE 4*, *GA20ox4*; *GIBBERELLIN INSENSITIVE DWARF 1*, *GID1s*), auxin (*YUCCAs*, *IAAs*), and cytokinin (*ISOPENTENYL TRANSFERASE 9*, *IPT9*; *Type-B ARABIDOPSIS RESPONSE REGULATOR*, *B-ARRs*) (Fig. 3 *E*, *G*, and *H*). Furthermore, several well-known genes involved in meristem maintenance and proliferation, such as *ASYMMETRIC LEAVES1 (AS1)*, *YABBY1* (*YAB1*), *KNOTTED1-LIKE HOMEOBOX GENE 3* (*KNAT3*), and *CLV3/ESR-related genes* (*CLE16*), were down-regulated in these mutants (Fig. 3 *E* and *I*). The broad dysregulation of genes linked to growth cessation, phytohormone pathways, and meristem dynamics in *AP2L/TOEL* mutants suggests that these TFs act as master regulators integrating multiple pathways to control meristem activity.

### *miR172-AP2L/TOEL* Mediates Growth Cessation Through Activation of *FT2* in the Leaf.

**Fig. 4.**
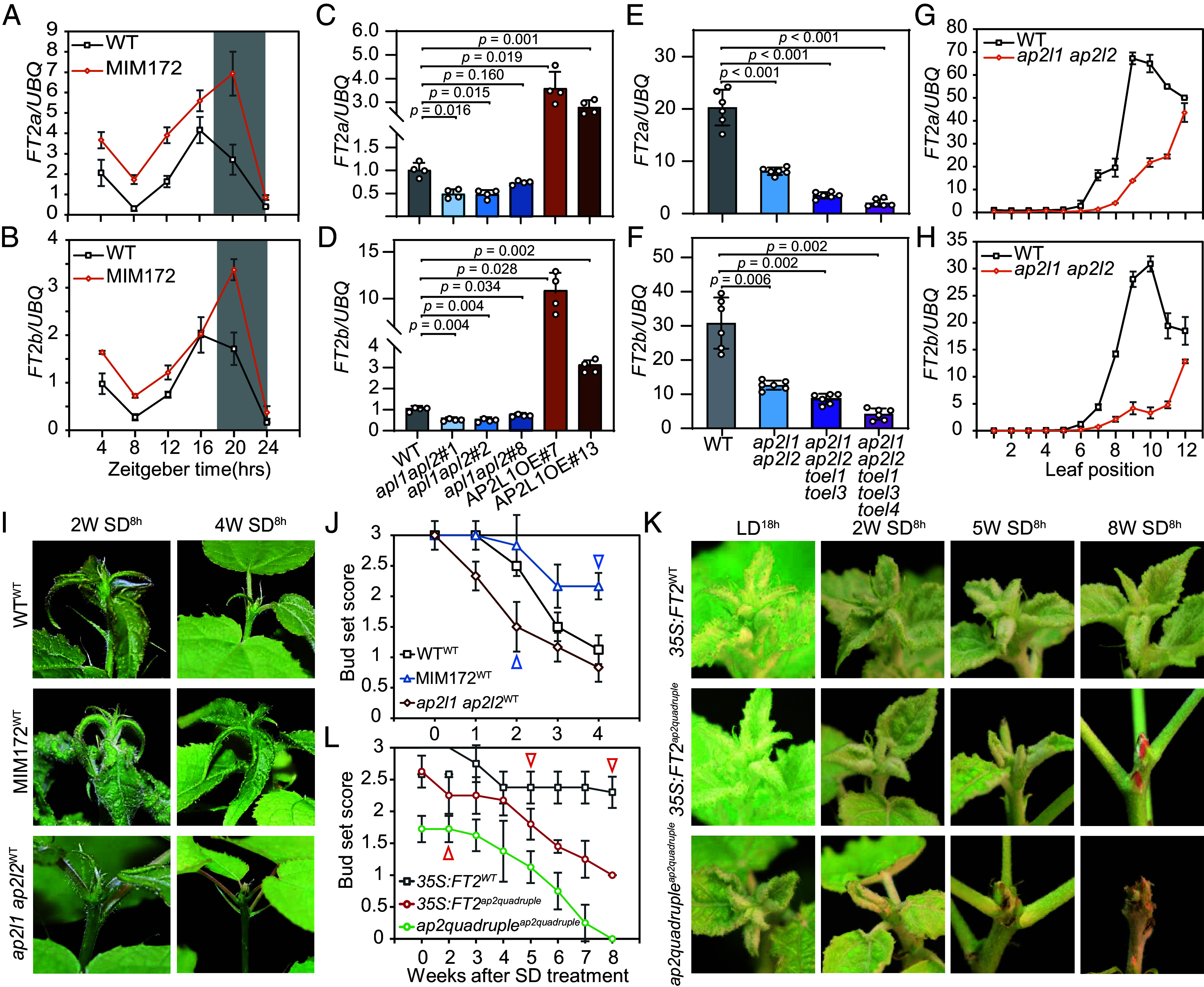
*AP2L/TOEL* inhibit growth cessation through activation of *FT2* in the leaf. (*A* and *B*) Diurnal expression patterns of *FT2a* and *FT2b* in mature leaves (leaf 9) of WT and MIMI172 plants grown under LD^18h^ conditions. Gray and white boxes represent night and day, respectively. (*C* and *D*) *FT2a* and *FT2b* expression in leaves of T89, *ap2l1 ap2l2* mutant, and rAP2L1OE lines. (*E* and *F*) *FT2a* and *FT2b* expression in leaves of T89, *ap2l1 ap2l2*, *ap2l1 ap2l2 toel1 toel3,* and *ap2l1 ap2l2 toel1 toel3 toel4* mutant lines. (*G* and *H*) Expression patterns of *FT2a* and *FT2b* in T89 and *ap2l1 ap2l2* in leaves of different ages. Leaves collected from the top to the bottom (leaf order, 1 to 12) in LD^18h^ conditions. The leaf samples used in (*A*–*F*) were taken from a mix of mature leaves (leaf 9 to leaf 11 counted from the top). All data shown in *A*–*H* represent mean values ± SE from three independent biological replicates. ZT, zeitgeber time. The significant differences between transgenes and WT or between combinations in (*C*–*F*) were determined by one-way ANOVA with the Games–Howell test. (*I* and *J*) Grafting assay of WT, MIM172, and *ap2l1 ap2l2*. Compared to WT control plants (T89 scions grafted on T89 stocks, T89^T89^, plants in superscript were used as scions for each graft), *ap2l1 ap2l2* ^T89^ plants ceased growth earlier, while MIM172^T89^ T89 plants delayed growth cessation. Shoot apices of representative grafts were taken at 2 and 4 wk after SD^8h^ treatment (*I*). (*J*) Bud set score of different grafts after transfer from LD^18h^ to SD^8h^ conditions. Data shown are mean values from four to six plants of each line. Error bars ± SE. Red triangles denote corresponding score values of shoot apices in (*I*). (*K* and *L*) Grafting assays of WT (717), *ap2l1 ap2l2 toel1 toel3* and *35S::FT2*. *ap2l1 ap2l2 toel1 toel3^ap2l1 ap2l2 toel1 toel3^*plants displayed strong defects in growth or completely ceased growth under LD^18h^ conditions. In comparison, *35S::FT2 ^ap2l1 ap2l2 toel1 toel3^* plants delayed growth cessation, in which the shoot apex did not stop growing until 5 wk of SD^8h^ treatment. While *35S::FT2*^717^ maintained growth even after 8 wk of SD^8h^. Shoot apices of representative grafts were taken at LD^18h^, 2, 5, and 8 wk after SD^8h^ treatment, respectively (*K*). (*L*) Bud set score of different grafts after transfer from LD^18h^ to SD^8h^ conditions. Red triangles denote corresponding score values of shoot apices in (*K*). Data shown in (*J* and *L*) are mean values from four to six plants of each line. Error bars ± SE.

In *Populus,* leaf-expressed *FT2* (two paralogs in hybrid aspen, *FT2a* and *FT2b*) is known as the key factor regulating the timing of growth cessation and bud set (9, 22). The *AP2L/TOEL* genes are also expressed in leaves (Fig. 3*A*). To investigate whether *AP2L/TOEL* mediates growth cessation through regulation of *FT2* in the leaf*, we* analyzed the expression of *FT2a* and *FT2b* in MIM172 leaves under LD^18h^ conditions. Compared to WT, both *FT2a* and *FT2b* displayed diurnal expression patterns, and were overall upregulated in MIM172 plants (Fig. 4 *A* and *B*). Consistently, both *FT2a* and *FT2b* were downregulated in *ap2l1 ap2l2* leaves, and upregulated in rAP2LOE leaves at the end of the day (ZT16) (Fig. 4 *C* and *D*). The expression levels of *FT2a* and *FT2b* decreased even more in *ap2l1 ap2l2 toel1 toel3* quadruple and *ap2l1 ap2l2 toel1 toel3 toel4* quintuple mutants (Fig. 4 *E* and *F*). *The miR172-AP2* module has been reported to regulate flowering downstream of *miR156* in *Arabidopsis* (23), and our previous studies have confirmed that *Populus miR156* regulates growth cessation in the age-dependent pathway (24). Thus, we also compared the expression pattern of *FT2* in different positioned leaves (with the youngest leaf at position 1) of *ap2l1 ap2l2* plants grown under LD^18h^ conditions. In contrast to what was reported in miR156eOE plants (24), both *FT2a* and *FT2b* were reduced in *ap2l1 ap2l2* plants compared to WT, with a delayed increase in expression (Fig. 4 *G* and *H*). These results suggest that *AP2L/TOEL* also have a role in the age-dependent *FT2* regulation.

Similar to *Arabidopsis* FT, FT2 protein is also a long-range mobile signal moving from leaf to shoot apex to mediate shoot growth (11). To validate that *FT2* acts downstream of *AP2L/TOEL* in the leaf, we performed grafting experiments, including T89^T89^, MIM172^T89^, and *ap2l1 ap2l2*^T89^ (plants in superscript were used as scions for each graft). Following transfer to SDs, shoot growth assessment revealed that MIM172^T89^ grafts exhibited delayed growth cessation compared to T89^T89^ controls, whereas *ap2l1 ap2l2*^T89^ grafts ceased growth earlier (Fig. 4 *I* and *J*). These observations suggest that the altered growth cessation timing in *MIM172* and *ap2l1 ap2l2* plants is likely to be caused by up- or down-regulation of *FT2*, respectively. We then conducted additional grafting experiments, including *ap2l1 ap2l2 toel1 toel3 ^ap2l1 ap2l2 toel1 toel3^*, *35S::FT2 ^ap2l1 ap2l2 toel1 toel3^* and *35S::FT2* ^WT^, to check whether FT2 can rescue the growth cessation defects in *ap2l1 ap2l2 toel1 toel3* mutant. The results showed that apices of *ap2l1 ap2l2 toel1 toel3* self-grafts initiated growth cessation even under LD^18h^ conditions and set bud already 5 wk after shifting to SD^8h^ conditions (Fig. 4 *K* and *L*), while *35S::FT2 ^ap2l1 ap2l2 toel1 toel3^* plants maintained active growth until after 5 wk of SD^8h^ treatment (Fig. 4 *K* and *L*). As a control, *35S::FT2* ^WT^ plants remained in active growth even after 8 wk of SD^8h^ treatments (Fig. 4 *K* and *L*). Although 3*5S::FT2 ^ap2l1 ap2l2 toel1 toel3^* plants displayed significantly delayed growth cessation compared to *ap2l1 ap2l2 toel1 toel3 ^ap2l1 ap2l2 toel1 toel3^* plants, both genotypes eventually formed buds after 8 wk of SD^8h^ treatment (Fig. 4 *K* and *L*). These results indicate that *FT2* overexpression can partially, but not fully, rescue the growth cessation defects in *ap2l1 ap2l2 toel1 toel3* mutants.

### Genome-Wide Identification of Direct Targets of AP2L.

**Fig. 5.**
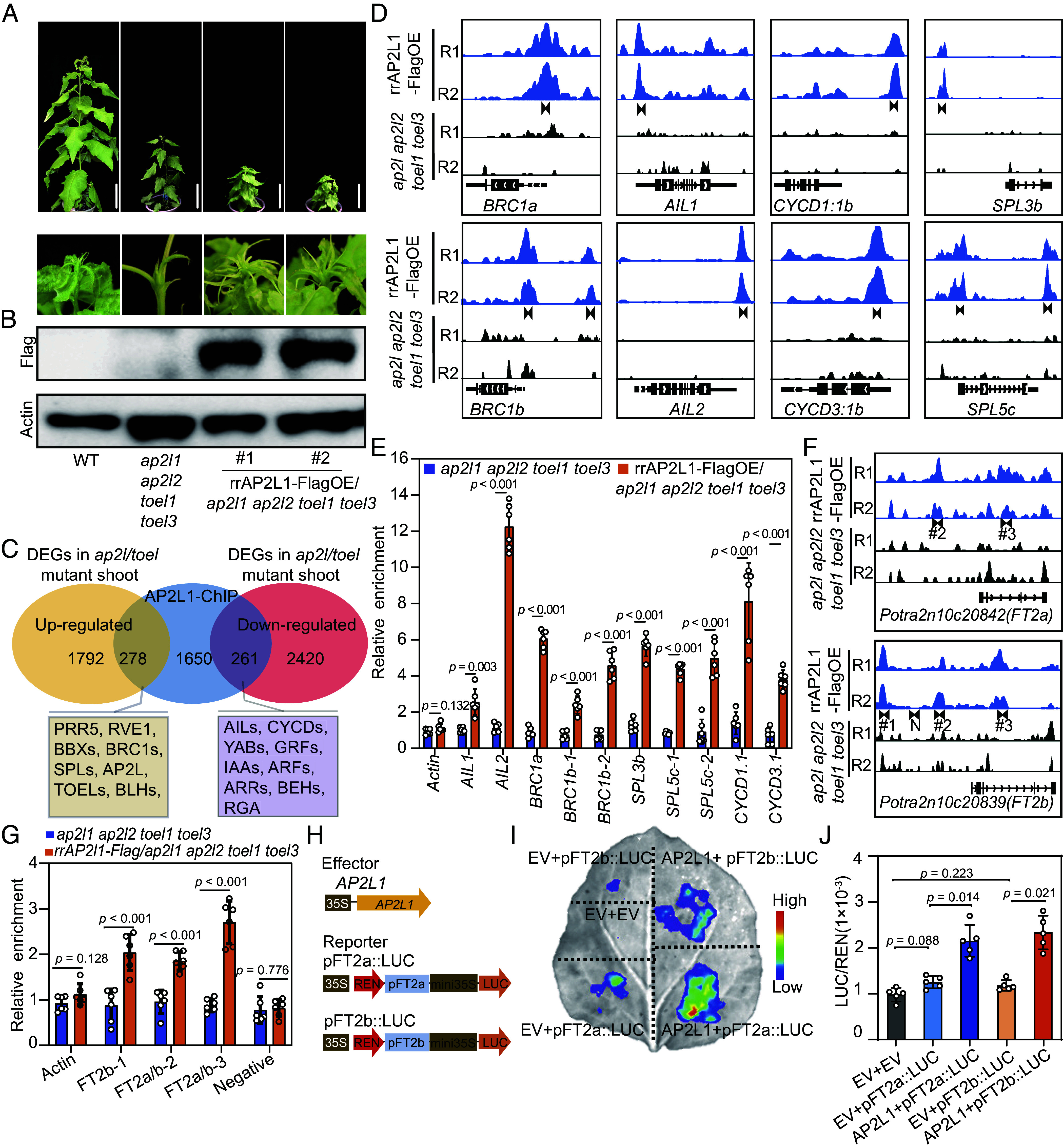
Genome-wide identification of direct targets of AP2L. (*A*) Morphologies of the complementary transgenic plants by overexpression of Flag-tagged *AP2L* (rrAP2L1-FlagOE) in *ap2l1 ap2l2 toel1 toel3* mutants. rrAP2L1-FlagOE can recover the apical growth defects of *ap2l1 ap2l2 toel1 toel3* mutants. (Scale bar, 10 cm.) (*B*) rrAP2L1 protein detection in the complemented plants using a Flag antibody. Actin was used as the internal reference. (*C*) Venn diagram showing the common genes between AP2L1 direct targets (*P* < 1 × 10^−3^) and DEGs identified from the *ap2l/toel* mutants identified in Fig. 3*B*. The names of corresponding representative genes are shown in the colored boxes. (*D*) Binding profiles of selected target genes are shown, with gene structures, names, and locus identifiers displayed below each panel. *Upper* panels show replicate experiments (R1, R2) from rrAP2L1-FlagOE lines, while *Middle* panels display corresponding profiles from *ap2l1 ap2l2 toel1 toel3* mutants. The enriched AP2L1 binding sites are labeled with black arrows and primers from the binding sites were designed and used for ChIP-qPCR. (*E*) ChIP–qPCR validation confirmed AP2L binding to all candidate genes shown in *D*. *ap2l1 ap2l2 toel1 toel3* quadruple mutants immunoprecipitated with anti-Flag antibody served as negative controls. DNA enrichment was quantified as the fold-change ratio of rrAP2L1-FlagOE related to *ap2l1 ap2l2 toel1 toel3* quadruple mutants. The DNA fragments of the Actin gene were used as controls. Data shown are mean values from three biological replicates. Error bars ± SE. (*F*) The AP2L1 binding profile on *FT2a* and *FT2b*. (*G*) ChIP–qPCR assay validation of AP2L binding to the *FT2a* and *FT2b* promoter and intron loci shown in *F*. The details of the binding profile and ChIP–qPCR assays are the same as in *D* and *E*. The significant differences between rrAP2L1-FlagOE and the *ap2l1 ap2l2 toel1 toel3* control in (*E* and *G*) were determined by unpaired *t* tests with Welch’s correction. (*H*) Schematic representation of the effector and reporter constructs used for transient expression assays in (*I* and *J*). (*I* and *J*) Transient expression assay in *Nicotiana benthamiana* leaves demonstrated that AP2L functions as a transcriptional activator of both *FT2a* and *FT2b*. Data were normalized to the internal control *35S*::*REN*. All data are means ± SE (n = 3). One-way ANOVA with the Games–Howell test was used to determine the significance of differences between combinations. EV, empty vector.

To further unveil the mechanisms of *AP2L/TOEL* in growth cessation, we overexpressed *rrAP2L1* (both miR172 and gRNA resistant version) fused to a 3xflag epitope tag (rrAP2L1-FlagOE) and complemented the *ap2l1 ap2l2 toel1 toel3* quadruple mutant. Two independent lines were obtained (Fig. 5 *A* and *B*). Both lines could recover the growth defects in the *ap2l1 ap2l2 toel1 toel3* quadruple mutant and displayed similar growth phenotypes to rAP2L1OE plants including short stature and shorter internode distances (compare Fig. 2*A* and Fig. 5*A*). With this material, chromatin immunoprecipitation-sequencing (ChIP-seq) was applied to identify AP2L1 targets. A total of 2,118 putative *AP2L1* target genes were identified in both two trials (Dataset S3*A*). Of them, 278 and 261 genes were found to overlap with up-regulated and down-regulated DEGs in *AP2L/TOEL* mutant shoot apices, respectively, suggesting that these genes may well be directly regulated by *AP2L1* (Fig. 5*C* and Dataset S3 *B* and *C*). This result also suggests that, similar to AP2 in *Arabidopsis* (25), *AP2L1* may have dual molecular roles and functions as both a transcriptional activator and a repressor. Many genes encoding TFs or key regulators that are involved in the regulation of growth cessation (*BRC1s, SPLs, AILs, CYCDs*), SAM activity maintenance (*GRFs, YABs,* and *KNATs*), phytohormone signals (*GID1s, IAAs, ARFs,* and *ARRs*), and biotic and abiotic responses (*MYB14, MYB55, MYB106, WRKY11, WRKY18,* etc.), were found to be direct targets of *AP2L1* (Fig. 5*C* and Dataset S3 *B* and *C*). These results suggest that AP2L*/*TOEL act bifunctionally as a hub to maintain SAM activity.

We then focused on representative genes that have been reported to regulate seasonal growth cessation (13, 15, 23), including *BRC1a/b, SPL3/5, AIL1/2,* and *D-type cyclins* (Fig. 5*D*)*. BRC1s* have been found to act both downstream and in parallel with *FT2* (15); *SPL3/5* are direct repressors of *FT2* in an age-dependent manner (23); *AIL1/2* and *D-type* cyclins are final executors in facilitating cell cycle progression in the shoot apex (13). ChIP with qPCR (ChIP-qPCR) assays confirmed that *AP2L1* interact with all these loci (Fig. 5*E*). We also identified two and three significant enrichments of AP2L1 binding sites in the *FT2a* and *FT2b* genomic regions, respectively (Fig. 5*F*). ChIP-qPCR assays confirmed the AP2L1 enrichment at the *FT2* loci (Fig. 5*G*). Further dual-luciferase reporter assays showed that AP2L1 can significantly activate the expression of *FT2a/b* (Fig. 5 *H*–*J*). These results showed that AP2L*/*TOE associate with *FT2* promoter to directly activate its expression.

### The *miR172-AP2L/TOEL* Module Is Involved in Age-Dependent Growth Cessation by Acting Downstream of the *miR156*-*SPL* Regulatory Module.

**Fig. 6.**
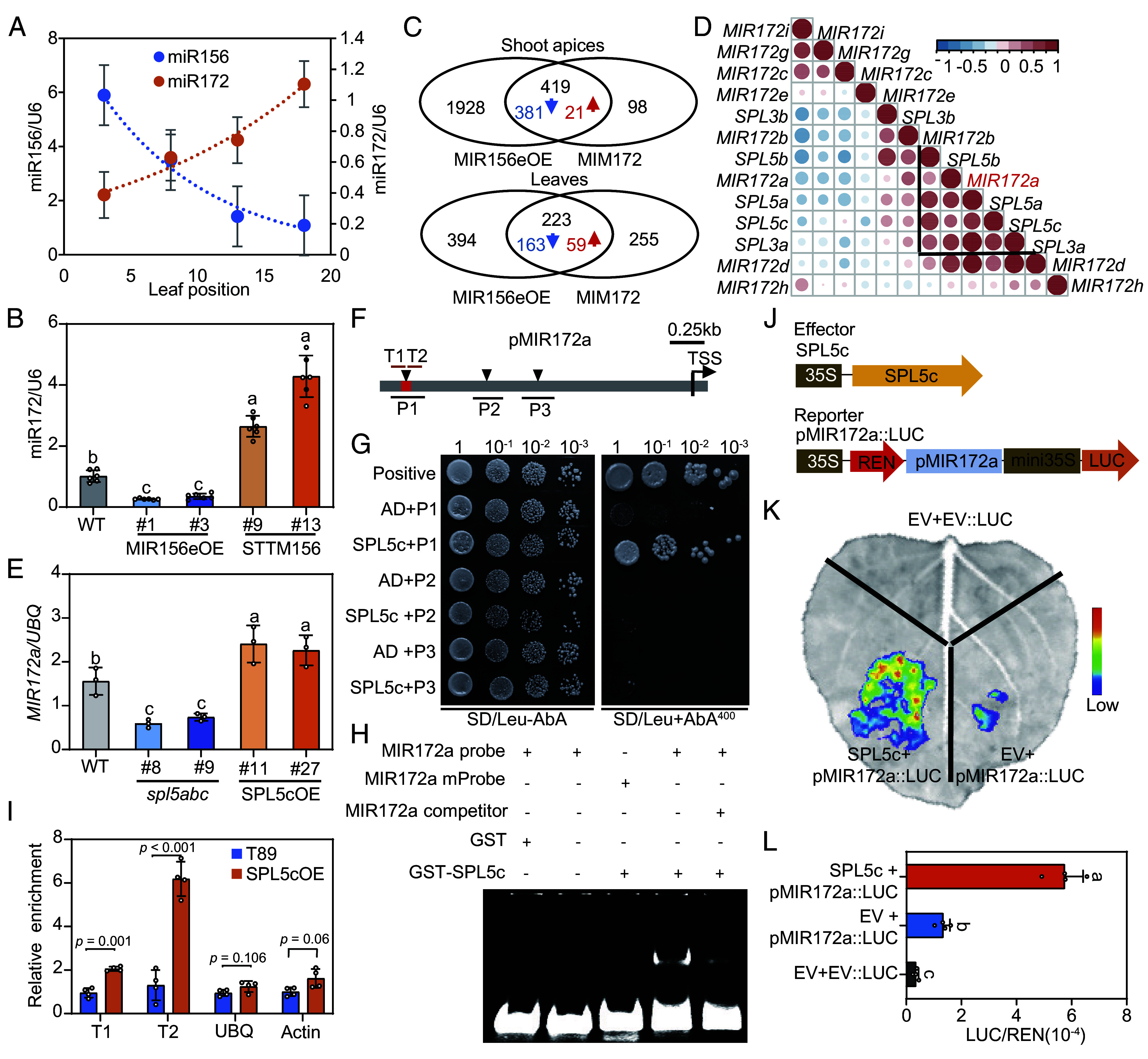
*miR172-AP2L/TOEL* acts downstream of the miR156-SPL module. (*A*) Expression patterns of *miR156* and *miR172* in hybrid aspen leaves. Leaves from the most juvenile bottom leaf (“1”) to the more mature top leaf (“20”) were collected in LD^18h^ conditions. (*B*) Expression levels of *miR172* in WT, miR156eOE, and STTM156 plants. (*C*) Venn diagram showing the overlap of DEGs identified from miR156eOE and MIM172 leaves and shoot apices, respectively. Numbers in blue and red represent common down- and up-regulated genes in miR156eOE and MIM172 plants. (*D*) Correlation analysis of *SPL3/5* clade genes and *MIR172* primary transcripts based on the year-round expression patterns of these genes. *MIR172a* displayed higher correlations with *SPL3/5*. (*E*) Expression levels of *MIR172a* in WT, SPL5cOE, and *spl5abc* plants. Leaf samples for RNA quantification in (*B*) and (*E*) were taken from tissue culture plants of each transgenic line under LD^18h^ conditions. Data in (*A*, *B*, and *E*) are mean values from three biological replicates. Error bars ± SE. *U6* or *UBQ* was used as reference genes. (*F*) Schematic diagram of the SPL5c binding motives (black triangles) in the *MIR172a* loci, the amplicon locations for the ChIP-qPCR analysis (T1, T2), the fragments used for the Yeast one hybrid assay (P1 to P3) and the probes for the EMSA (red box). (*G*) Yeast one-hybrid assay demonstrating direct binding of SPL5c to the *MIR172a* promoter region. (*H*) EMSA of GST-SPL5c binding to the *MIR172a* promoter. *MIR172a* probe containing the GTAC motif was labeled by DY680. The DNA–protein complex formation was abolished when using probes with mutated SPL-binding sites. Binding specificity was further confirmed through competition assays with excess unlabeled wild-type *MIR172a* probes. (*I*) ChIP-qPCR analysis identified specific MIR172a chromatin regions bound by SPL5c in plants harvested under LD^18h^ conditions. Enrichment values were normalized to the *UBQ* gene control amplicon. The significant differences between SPL5Coe and WT control were determined by unpaired *t* tests with Welch’s correction. (*J*) Schematic diagrams of effector and reporter constructs used for transient expression assays in (*K* and *L*). (*K* and *L*) Transient coexpression in *N. benthamiana* leaves demonstrates that SPL5c transcriptionally activates *MIR172a* expression. Data are normalized to the internal control *35S*::*REN*. All data are means ± SE (n = 3). EV, empty vector. One-way ANOVA with Tukey’s multiple comparison test was used to determine the significance of differences among WT and transgenics lines or among different combinations in (*B*, *E*, and *L*).

In annual plants, the *miR156/miR172* module controls vegetative phase change and reproductive competence during age-dependent flowering regulation (22). As plants age, a gradual decline of *miR156* abundance occurs while, in contrast, there is a steady increase in the levels of *miR172* (22). The same can be seen in *Populus* leaves of different ages (Fig. 6*A*). As *miR156* has been found to play key roles in age-dependent growth cessation (23), we speculated that *miR172* regulates growth cessation downstream of *miR156* in this age-dependent pathway. As predicted, the expression level of the mature *miR172* was down-regulated in miR156eOE and up-regulated in STTM156 (suppressing the activity of *miR156*) plants (Fig. 6*B*). To check the relationship between the *miR156* and *miR172* pathways, we collected leaves and shoot apices from miR156eOE, MIM172 transgenic, and WT plants under LD^18h^ conditions for transcriptome analysis. In the shoot apex, 80 % of DEGs (419 out of 517) identified in MIM172 were included in the miR156eOE DEGs with the same expression trend compared to WT (Fig. 6*C*). This result suggests that in the shoot apex, *miR172* regulation of growth cessation is largely dependent on the *miR156* pathway. In contrast, only 46.7% (223 out of 478) MIM172 DEGs overlapped with miR156eOE DEGs in leaves, suggesting that, besides the *miR156* pathway, other signaling pathways in leaves are important for the function of *miR172* (Fig. 6*C*). In annual plants, the *miR156* targets, the *SPL* genes, have been found to directly associate with the *MIR172* loci to activate their expression (22). Our previous studies have confirmed that the *SPL3/5* genes play major roles in growth cessation regulation (23). To find the possible downstream *MIR172* gene loci, we made an expression pattern correlation analysis among five *SPL3/5* genes and eight *MIR172* primary transcripts based on the year-round RNA-seq data. The result showed that *MIR172a* displayed the highest expression correlation with the *SPL3/5* genes (Fig. 6*D*). Moreover, the expression of *MIR172a* was down-regulated in *spl5abc* and up-regulated in SPL5cOE plants (Fig. 6*E*). Three putative *SPL5c* binding motifs (GTAC) in the *MIR172a* promoter region were identified (Fig. 6*F*). Further yeast one-hybrid (Y1H) assays showed that SPL5c could bind to the P1 promoter region of *MIR172a*, but not to two other sites (Fig. 6*G*). We then performed electrophoretic mobility shift assays (EMSAs) using a probe designed based on the P1 region. The results showed that GST-SPL5c bound to the probe (Fig. 6*H*), but not when the SPL5c-binding site was mutated or in the presence of unlabeled competitor probes (Fig. 6*H*). We also performed ChIP-qPCR assay using myc-tagged SPL5cOE transgenics. The result showed that there were two- and sixfold enrichment in this region with two pairs of primers located near the binding site (Fig. 6*I*). Therefore, both in vitro and in vivo data confirm direct binding of SPL5c to the *MIR172a* promoter. Furthermore, dual-luciferase reporter assays showed that SPL5c can significantly activate the expression of *MIR172a* through this direct binding (Fig. 6 *J*–*L*).

### *AP2L/TOEL* Regulates Growth Cessation Through Feedback Regulation of the *miR156/SPL* Module.

**Fig. 7.**
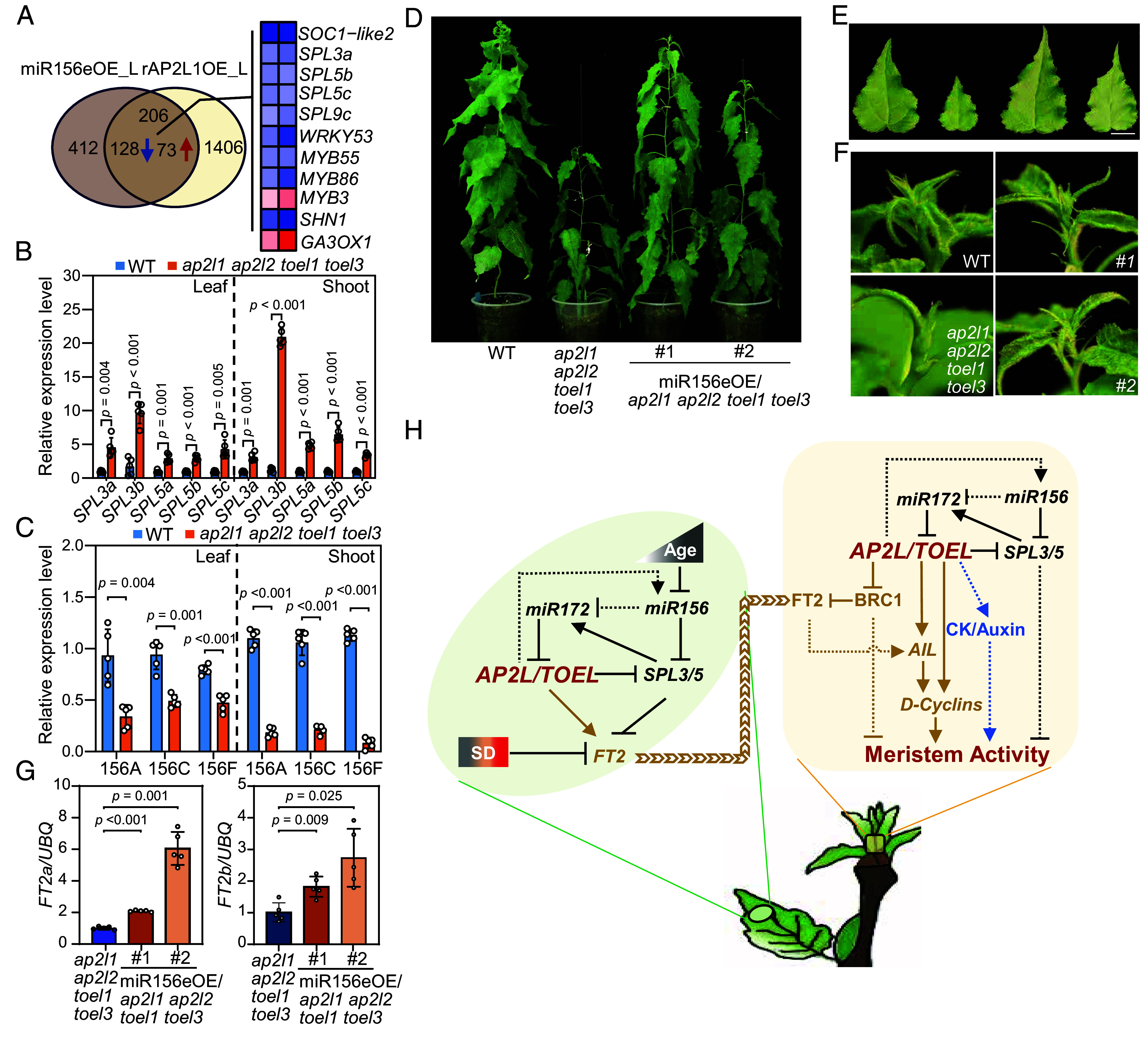
*AP2L/TOEL* regulates growth cessation through feedback regulation of the *miR156/SPL* module. (*A*) Venn diagram showing the common DEGs identified from miR156eOE and rAP2L1OE leaves, including *SPL* genes. Log2Foldchange values were used to for heatmap analysis. (*B* and *C*) Expression analysis of *SPL3/5* clade genes and primary transcripts of *miR156* in WT and *ap2l1 ap2l2 toel1 toel3* plants in leaves and shoot apex, respectively. The significant differences between transgenes and WT were determined by unpaired t-tests with Welch’s correction. (*D*–*F*) Plant morphologies of WT, *ap2l1 ap2l2 toel1 toel3* and MIR156eOE/*ap2l1 ap2l2 toel1 toel3* plants. Overexpression of *MIR156e* in *ap2l1 ap2l2 toel1 toel3* mutants partially rescues plant height (*D*), leaf size (*E*), shoot apical activity and trichome density (*F*). The youngest fully expanded leaves (10–12) from the shoot apex were selected to measure the leaf morphology in (*E*). The recovered activity of shoot apices are highlighted with red triangles in (*F*). (*G*) Expression levels of *FT2a* and *FT2b* in leaves of *ap2l1 ap2l2 toel1 toel3* and miR156eOE/*ap2l1 ap2l2 toel1 toel3* plants. The significant differences between transgenes and WT were determined by one-way ANOVA with the Games–Howell test. (*H*) Molecular network of *AP2L/TOEL* in the control of growth cessation in *Populus*. *AP2L/TOEL* inhibit growth cessation both in the leaf and shoot apex. In the leaf, *AP2L/TOEL* directly promote *FT2* expression, which in turn moves to shoot apices to maintain vegetative growth. In the shoot apex, *AP2L/TOEL* maintain shoot activity mainly through two pathways, one is bifunctional and directly represses *BRC1* or activate *AIL* and D-type cyclin genes in both *FT2* dependent and independent pathways (brown lines). In another pathway, *AP2L/TOEL* maintain shoot activity through phytohormone signals, such as CK and auxin, which is worthy of further investigation (blue lines). *AP2L/TOEL* genes are also involved in age-dependent growth cessation by feedback regulation of the *miR156-SPL3/5* module in both leaf and shoot apex (black lines). Solid lines indicate direct molecular interactions or experimentally validated effects on growth processes. Dotted lines denote putative regulatory relationships requiring further characterization.

When we compared the DEGs identified in miR156eOE and rAP2L1OE transgenic leaves collected under LD^18h^ conditions, we found that a large proportion of DEGs in miR156eOE leaves overlapped with DEGs in the rAP2L1OE transgenics, including the *SPL3/5* genes (Fig. 7*A*). Consistently, all five *SPL* genes from the *SPL3/5* clade were up-regulated in *ap2l1 ap2l2 toel1 toel3* mutants both in leaf and shoot apex samples (Fig. 7*B*). Moreover, the expression of three primary transcripts of *miR156* (*MIR156A/C/F*) were down-regulated in *ap2l1 ap2l2 toel1 toel3* mutants (Fig. 7*C*). These data led us to speculate that *AP2L/TOEL* may control growth cessation, at least partly, through feedback regulation of the *miR156/SPL* module. To confirm this, we transformed the miR156eOE construct into *ap2l1 ap2l2 toel1 toel3* plants, and two independent lines with high expression of *miR156* were obtained (*SI Appendix*, Fig. S13). When potted in the greenhouse, both transgenic plants partially recovered the defects observed in *ap2l1 ap2l2 toel1 toel3* mutants, including height, leaf size, and trichomes (Fig. 7 *D*–*F*). Moreover, the shoot apex in miR156eOE/*ap2l1 ap2l2 toel1 toel3* plants became more active and delayed growth cessation compared to *ap2l1 ap2l2 toel1 toel3* plants (Fig. 7*F*). This may partially be due to the up-regulation of *FT2a* and *FT2b* in miR156eOE/*ap2l1 ap2l2 toel1 toel3* plants (Fig. 7*G*). Additionally, we found that AP2L1 associates with the promoter region of *SPL3b* as well as with the promoter and 3′-UTR regions of *SPL5c* (Fig. 5*D*). However, no binding site was found in any *MIR156* gene locus. These results suggest that AP2L represses the expression of *SPL3b/SPL5c* directly, while it induces *MIR156*s indirectly through an unknown pathway. Taken together, these results strongly suggest that *AP2L/TOEL* regulates growth cessation through feedback regulation of the *miR156/SPL* module, integrating the *miR156* and *miR172* pathways in the regulation of *FT2*.

## Discussion

Cessation of growth prior to the onset of winter is a key developmental transition, the time of which is critical to the survival of perennial plants in boreal forests (26). Our study reveals that in *Populus* trees, the activity of *AP2L/TOEL* genes is critical to maintain vegetative growth and prevent premature growth cessation, integrating signals from both leaves and shoot apices (Fig. 7*H*). In leaves, AP2L/TOEL inhibit growth cessation by directly activating *FT2*, which in turn moves to shoot apices to sustain vegetative growth. Notably, AP2L/TOEL are the most potent direct activators of *FT2* expression identified to date. Since the transcriptional levels of *AP2L/TOEL* are not photoperiod-sensitive (*SI Appendix*, Fig. S14), and *CONSTANS* orthologs seem to play a minor role in *FT2* regulation (27, 28), it is possible that the photoperiodic regulation of *FT2* expression is rather occurring through the regulation of *FT2* repressors, such as CDFs (28), SPLs (24), and PIFs (29), possibly acting through competitive binding with the AP2L/TOEL activators. However, at this stage we can not exclude that there is also a photoperiodic regulation of the miR172-AP2L/TOEL regulon through the regulation of AP2L/TOEL protein translation and stability. The precise molecular mechanism remains unclear and warrants further investigation. Besides its critical activity in regulating *FT2* expression in the leaf, in the shoot apex, *AP2L/TOEL* maintain meristem activity through two major pathways: First, through a bifunctional regulation, repressing *BRC1* or activating *AIL* and D-type cyclin genes in both *FT2-*dependent and -independent manners (Fig. 7*H*, brown lines); and second, through modulation of phytohormone signaling, particularly auxin and cytokinin (CK) signaling (Fig. 7*H*, blue lines). Intriguingly, CK and auxin are two major phytohormones that have been reported to control GPA in annual plants, yet how *AP2L/TOEL* mediate their effects on shoot activity remains poorly understood and merits further study. Additionally, we found that the miR172-AP2L/TOEL module is involved in age-dependent growth cessation by acting downstream of the miR156-SPL regulatory module (Fig. 7*H*, black lines). This module indirectly influences growth cessation via feedback regulation of the *miR156-SPL3/5* pathway in both leaves and shoot apices. Together, these findings underscore the multifaceted role of AP2L/TOEL in coordinating diverse pathways to regulate SAM activity and growth. Our work advances the understanding of how perennial plants control seasonal growth cessation in response to endogenous and environmental cues (Fig. 7*H*).

In *Arabidopsis*, two major pathways have been proposed to control GPA: age-dependent and seed-dependent signaling (30). Among these, AP2 and AP2-Like TFs have been identified to play a central role in the GPA process by integrating both pathways and delaying the onset of GPA through sustained expression of the *WUSCHEL* (*WUS*), a key stem cell identity gene (31). The *miR172-AP2* module is known to regulate developmental phase transitions via the age-dependent pathway (23, 32), and recent studies indicate that it cooperates with the MADS-box TF FUL to control GPA timing under age-dependent cues (5). CK is a major phytohormone regulating GPA. In IM, repression of CK signaling is required for proliferative arrest (33). In SAM, CK maintains stem cell populations by activating *WUS* expression through ARRs or by modulating cell-cycle components such as CYCB, CYCD, and CDKs (34). Notably, AP2 enhances CK responses in the SAM by repressing negative regulators of CK signaling (7). Beyond CK, auxin also influences GPA. Developing fruits export auxin, which disrupts polar auxin transport and triggers IM arrest (35), though the underlying molecular mechanisms remain unclear. Additionally, GPA is associated with the accumulation of ABA-responsive genes, leading to its classification as a dormancy state. Intriguingly, several bud dormancy-related genes function downstream of the *AP2* pathway (7). Strikingly, all these GPA-associated pathways were enriched in our AP2L/TOEL mutants in *Populus*, suggesting that AP2 orthologs regulate growth cessation through conserved mechanisms similar to those in *Arabidopsis*. To some extent, both GPA and seasonal growth cessation represent a transient meristematic arrest. However, the ultimate fate of the SAM differs significantly between species: In monocarpic plants, the whole plant will precede senescence and die after GPA, whereas in polycarpic trees, shoots will enter dormancy after growth cessation. How these seemingly conserved pathways diverged functionally between monocarpic and polycarpic plants remains an open question for future research.

*Populus* contains six *AP2L/TOEL* genes, and our genetic data indicate that these genes function redundantly, particularly in the control of growth cessation control. The impact on growth cessation becomes pronounced with increasing number of mutations (Fig. 2 *J*–*L*), as observed in *AP2L/TOEL* quadruple, quintuple, and sextuple mutants, which exhibited a complete growth arrest already in tissue culture (Fig. 2*J*), indicating the necessity of *AP2L/TOEL* for SAM activity maintenance. While these genes show functional redundancy in the regulation of growth cessation, it is possible that there are distinct roles among the AP2L/TOEL genes in other biological processes. For instance, rTOEL1OE and rTOEL3OE plants exhibit significant defects in leaf development (Fig. 2*B*), whereas rAP2LOE plants display normal leaf development compared to WT plants. Future studies should explore the individual functions of these genes through single gene mutations to better understand their specific roles.

The integrated analysis of genome-wide direct binding and global gene expression changes indicates that *AP2Ls* in *Populus* may function as a bifunctional TF, a characteristic consistent with studies on *AP2* in *Arabidopsis* (25). The underlying mechanisms governing this bifunctional feature of *AP2Ls* remains largely unknown, but it is plausible that AP2Ls may interact with cofactors that modulate its transcriptional activation or repression roles, a phenomenon observed in other TFs (36). *AP2L* genes have been documented as repressors of the flowering transition in various species (37–39). In particular, the *Arabidopsis* AP2-like proteins, TOE1, and SMZ, have been shown to bind to *FT* promoter sequences and directly repress its expression (39). In contrast, we show here that in *Populus*, AP2L regulates growth cessation by directly activating *FT2*, which has dual roles in inhibiting growth cessation and promoting flowering (9). This divergence in gene function between *Populus* and *Arabidopsis* highlights a common theme in growth cessation regulation in trees, where molecular pathways may have opposite functions to the established flowering pathways from *Arabidopsis*, such as the roles of the *miR156/SPL* (24) and *PHYB/PIF8* (29) modules. The contrasting effects of these genes in *Populus* show how annual plants and perennial trees have evolved distinct mechanisms to integrate these modules into diverse developmental processes. This is further emphasized by our finding that the same *AP2L/TOEL* genetic pathway that in annual plants is controlling GPA after flowering and fruit set, in perennial trees control the annual growth cessation and meristem arrest occurring at the end of the growing season. This is a remarkable extension of the finding that the same genetic pathways that are controlling flowering in annual plants, involving genes like *LATE ELONGATED HYPOCOTYL 2* (*LHY2*), *GIGANTEA* (*PtGI*), *ELONGATED HYPOCOTYL 5* (*PtoHY5a*), *FT2,* and *LAP1*, are controlling the regulation of growth cessation and bud set in trees (10, 13, 29, 40–43). This again shows how in plants with different life histories the same regulatory modules can be used to regulate completely different developmental processes that are sharing similar basic mechanisms.

## Materials and Methods

Plant materials, plant phenotyping, grafting assay, vector construction, genetic transformation, transgene phenotyping, ChIP assay, bioinformatic analysis, RNA-sequencing, RNA quantification, yeast one-hybrid assay, EMSA, and dual luciferase transcriptional activity assay are described in detail in *SI Appendix*, *Materials and Methods*. Primer sequences are listed in Dataset S4.

## Supplementary Material

Appendix 01 (PDF)

Dataset S01 (XLSX)

Dataset S02 (XLSX)

Dataset S03 (XLSX)

Dataset S04 (XLSX)

## Data Availability

All study data are included in the article and/or supporting information.
