# Supplementary material for: Genetic control of seasonal meristem arrest in trees
Source: Proc Natl Acad Sci U S A. 2025 Nov 26;122(48):e2505641122. doi: 10.1073/pnas.2505641122 (PMC12685071; doi:10.1073/pnas.2505641122)
Supplement: Supplementary file 1 — Appendix 01 (PDF) [file pnas.2505641122.sapp.pdf]

Supporting Information for

Genetic Control of Seasonal Meristem Arrest in Trees

Jun Wang, Xiaoli Liao, Zhihao Wu, Shashank Sane, Shaopeng Han, Qihui Chen, Xueping Shi, Xiaokang Dai, Maria Klintenäs, Ove Nilsson and Jihua Ding

Corresponding authors: Ding, J and Nilsson, O

Email: jihuading@mail.hzau.edu.cn, Ove.Nilsson@slu.se

This PDF file includes:

Supplementary Materials and Methods
Figures S1 to S13
Tables S1
Dataset S1 to S4
SI References

Supplementary Materials and Methods

Plant materials, growth conditions and growth cessation phenotyping. Hybrid aspen *P. tremula x P. tremuloides* (clone T89) or hybrid poplar *P. tremula x P. alba* (INRA717-1B4) were used as wild type controls and for genetic transformation (1). Wild type and transgenic plants were grown in controlled chambers at 22°C/20°C (day/night) in long days (18 h day length; LD^{18h}) or short days (8 h day length; SD^{8h}). For plant growth cessation induction and bud set scoring, plants were grown in a growth chamber for approximately 8 weeks in LD^{18h} conditions, and then shifted to SD^{8h} conditions. Scoring started after transfer to SD^{8h} conditions. For scoring of bud break, after 10 weeks of SD^{8h} treatment, plants were transferred to a cold room with short days (SD^{8h}, 4°C) for 10 weeks and returned to warm LD^{18h} conditions. Growth cessation and bud break were scored according to the standard described in Fig. S3, and as well as previously described (2). Two or three independent and representative transgenic lines and 6-8 plants of each line were analyzed.

Generation of vector constructs and transformation. The artificial target mimic construction for *miR172* was kindly provided by the Weigel lab (3). For the MIR172bOE vector construction, a 242 bp fragment of *MIR172b* from hybrid poplar was amplified and cloned into pDONR207 (Invitrogen, USA). The MIR172bOE fragment was then ligated into the destination vector pK2GW7 driven by the constitutive cauliflower mosaic virus *35S* promoter (35S) (4). To generate miR172-resistant forms of the *AP2L1, TOEL1* and *TOEL3* CDSs, we substituted the miR172 target sequence with a synonymous mutant sequence while still maintaining an unchanged amino acid translation sequence (5) (Fig. S7A). The modified sequences were synthesized (GenScript Corporation, IA) and cloned into the destination vector pK2GW7 to generate the vectors rAP2L1OE, rTOEL1OE and rTOEL3OE, respectively. We generated rrAP2L1-FlagOE by fusing the flag epitope tag to the C-terminal of AP2L1, and also substituted the gRNA target sites of *AP2L1* with synonymous mutant sequences against the gRNA recognition sites in the *ap2l1 ap2l2 toel1 toel3* mutant. *ap2l1 ap2l2*, *toel1 toel2* and *toel3 toel4* knockout constructs were generated with the golden gate-based

GreenGate cloning system (6). The detailed procedures for vector construction have been described previously (7). All these constructs were individually transformed into hybrid aspen or hybrid poplar using *Agrobacterium tumefaciens*-mediated gene transfer according to standard protocols (8). *ap2l1 ap2l2 toel1 toel3* quadruple, *ap2l1 ap2l2 toel1 toel3 toel4* quintuple, and *ap2l1 ap2l2 toel1 toel2 toel3 toel4* sextuple lines were generated by co-transforming *toel1 toel2* and *toel3 toel4* knockout constructs into the *ap2l1 ap2l2* double mutant line #8.

Mutant screening. For genotyping of base-edited trees, we first amplified the target sites followed by Sanger sequencing to determine the editing efficiency. If they were edited, we genotyped them using the Hi-TOM platform to identify the mutations (9) (http://hi-tom.net/hi-tom/). Briefly, DNA was extracted from leaf tissues of wild type and transgenic plants using the CTAB methods (10). Target genomic regions were amplified by PCR and barcoded using Hi-TOM primers (9). PCR products were verified by gel electrophoresis and purified by QIAQuick PCR Purification Kit (QIAGEN). The purified PCR products were sequenced by next generation sequencing (NGS) on an Illumina HiSeq2500 sequencing platform. The NGS data were then analyzed with CRISPRMatch (11) and CRISPResso2 (12). To confirm that both parental alleles had been amplified and mutated in these sites, distinct allele-specific SNPs around the gRNA target region were used as parental allele markers. Primers used for construct generation are listed in Dataset S4.

Grafting assay.

All wild type and transgenic plants used for grafting were first grown under LD^{18h} for 6 weeks. Scions contained one lateral bud, approximately 2 cm in length, while the rootstock was decapitated ca 10 cm below the apex and kept with its leaves. All grafts were transferred to SD^{8h} after 3 weeks of grafting when the scion buds sprouted. 5-8 plants per graft combination was used for further analysis.

ChIP assay and ChIP-seq data analysis.

ChIP experiments and analysis were carried out following our previously described protocols (13, 14). Chromatin from both *ap2l1 ap2l2 toel1 toel3* and rrAP2L1-FlagOE plants was extracted from equally mixed shoot apices and leaves collected from plants grown for one month in LD18h conditions at ZT4. The fragmented chromatin was precipitated with antibodies against the Flag-tag (ABclonal, AE092, 5 μg ml^{-1}). The detailed procedures for ChIP-seq and ChIP–qPCR have been described previously (7). For ChIP-seq, two biological replicates for each of *ap2l1 ap2l2 toel1 toel3* and rrAP2L1-FlagOE ChIP–DNA were processed. The ChIP-seq libraries were then sequenced using a NovaSeq platform. ChIP-seq reads were mapped to the *P. tremula* genome reference v 2.2 (15). The detailed procedure for ChIP-seq analysis has been described previously (7). For ChIP–qPCR, the precipitated DNA were diluted and used as the PCR template. The isolated chromatin before precipitation was used as the input control. The UBQ amplicon was used as a negative control. All the primers for ChIP-qPCR are listed in Dataset S4.

RNA-Seq sampling and bioinformatics.

ap2l1 ap2l2, ap2l1 ap2l2 toel1 toel3 and *ap2l1 ap2l2 toel1 toel3 toel4*, rAP2L1OE, MIM172 and WT plants were grown in the greenhouse for approximately 4 weeks (LD^{18h}, 22°C). Leaf and/or shoot apices were collected from three independent plants for each genotype at ZT4, respectively. Total RNA was extracted using the CTAB-LiCl method and quantified using the Agilent 2100 Bioanalyzer (Agilent Technologies, Germany). Sequencing was performed on Illumina HiSeq2500 with paired-end 125 bp read length. The RNA-seq data were analyzed using a previously developed pipeline for quality control, read mapping, and expression quantification (16). Genes with FDR < 0.05 and fold change > 2 were selected as differential expression genes (DEGs) for further analysis.

RNA quantification.

Total RNA was extracted with the CTAB-LiCl method. cDNA synthesis was performed using the HiScript III 1st Strand cDNA Synthesis Kit (Vazyme, China) according to the manufacturer's

instructions. Quantitative real-time PCR analyses were carried out with a Roche LightCycler 480 II instrument, and the measurements were obtained using the relative quantification method (17). Results were normalized to the expression of *UBQ* RNA. To quantify the mature *miR156* and *miR172*, stem-loop quantitative PCR were performed as described previously (18). A complete list of primers used in real-time PCR analysis is presented in Dataset S4.

Yeast one-hybrid assay (YIH), electrophoretic mobility shift assay (EMSA) and dual-luciferase transcriptional activity assay.

YIH, EMSA and dual-luciferase transcriptional activity assay were performed as described previously (7). For Y1H, the truncated fragment of *MIR172a* promoter was amplified and cloned into the pABAi bait vector (Clontech, USA). The full-length coding sequence of *SPL5c* was cloned into the pGADT7 prey vector (Clontech, USA). The pABAi- *MIR172a* and pGADT7-SPL5c plasmids were transformed into the Y1HGold strain, independently. The bait colonies were cultured on SD/-Ura medium containing 0-500 ng/mL aureobasidin A (AbA) at 30°C for 3 days. After determining the most suitable AbA concentration, the prey vector pGADT7-SPL5c was transformed into the yeast strain harboring the bait vector. The transformed cells were grown on synthetic dropout medium (SD-Leu) plus 400 ng ml^{-1} AbA for 3 days. The pGADT7-p53 and pABAi-p53 vectors were transformed into the Y1HGold strain as a positive control.

For EMSA, the coding sequence of SPL5c was cloned into the pGEX6p-1 vector fused with GST and expressed in *Escherichia coli* strain BL21 (TransGen Biotech, China). Production of the recombinant proteins was induced using 0.5 mM isopropyl b-D-thiogalactoside (IPTG) with overnight incubation at 16°C and purified using glutathione Sepharose resin (GE Healthcare, USA) according to the manufacturer's instructions. DNA fragments containing the predicted SPL5c binding sites in the *MIR172a* genomic regions were synthesized and inserted into pGEM-T. After that, these probes were amplified by DY680-labeled or non-labeled primers. The EMSA assays

were performed as described previously (13). The DNA shift was detected using the ChemDoc XRS imaging system (Bio-Rad, USA).

For dual-luciferase transcriptional activity assay, the reporter construct was generated by cloning the promoter region of *MIR172a* (~ 2.5 kb), *FT2a* (~ 3 kb) or *FT2b* (~ 4 kb) into the pGreenII 0800-LUC vector to drive the expression of luciferase respectively. The effector vector was constructed by cloning the coding sequence of *SPL5c or AP2L1* into the pGreenII 62-SK vector (Clontech, USA). The effector and reporter constructs were co-transformed into *N. benthamiana* plants. The luciferase activities were measured using the GloMax® Discover System (GM3000, Promega, USA), and quantified with the Dual-Luciferase Assay Kit (E1910, Promega, USA) following the manufacturer's recommendations. The primers for Y1H constructions, dual-luciferase transcriptional activity assay constructions and probe sequences for EMSA are listed in Dataset S4.

Supplementary Figures

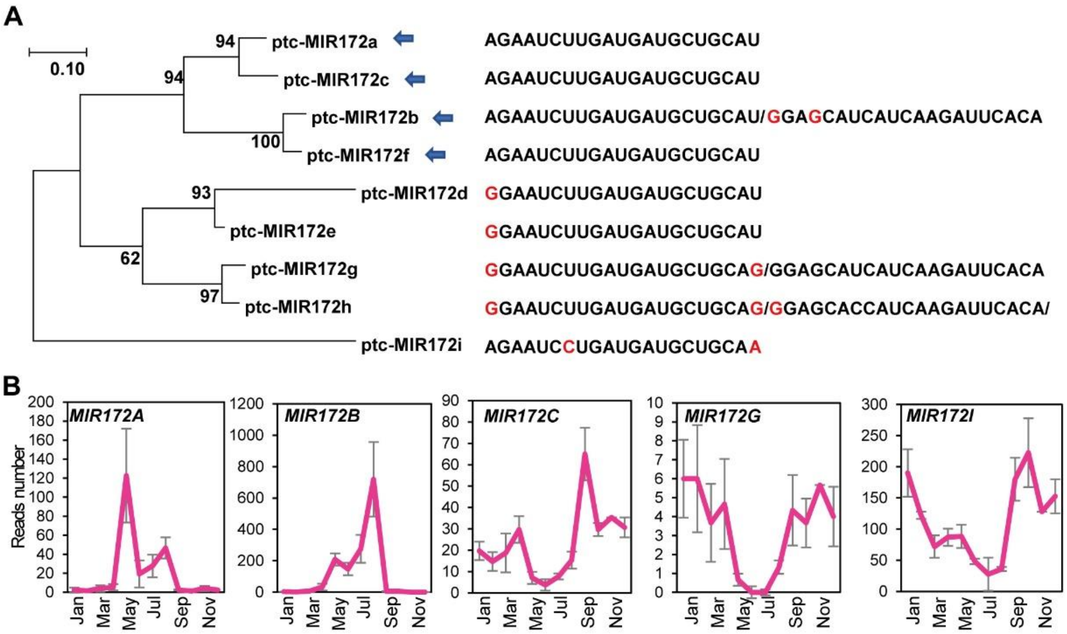

Fig. S1 Phylogenetic tree of *MIR172s* in *Populus trichocarpa* and their year-round expression patterns. (A) The phylogenetic tree was constructed with primary *MIR172* sequences exported from the miRbase website (http://www.*mirbase*.org/). The tree was constructed using MEGA 8 with the neighbor-joining method and 1000 bootstrap replicates. Bar, 0.10 changes per nucleotide position. Sequences on the right are the corresponding mature *miR172* sequences of each primary *MIR172*. The blue arrows represent the targets used for the design of the MIM172 constructions. (B) Year-round expression patterns of primary *MIR172* transcripts in aspen. Samples were taken from field-grown mature *Populus tremula* trees at 2 p.m. in the middle of each month over a course of one year. In May–August samples are from leaves, in September–April from terminal buds. Five of the primary *MIR172* transcripts were identified in this year-round transcriptome data.

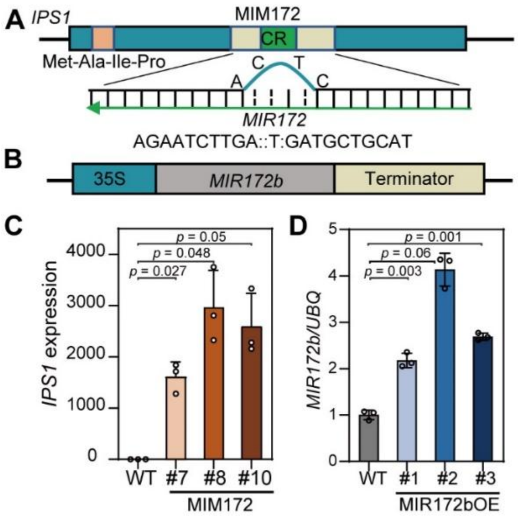

Fig. S2 Positive test of MIM172 plants and MIR172b over-expressors. (A) Schedule representing the MIM172 construction strategy. The *Arabidopsis miR399*-target-mimic *IPS1* gene was synthesized and the sequence complementary to *miR399* was replaced by a sequence complementary to *miR172* with a modification in the central sequence (TCTA to GAGT). (B) Structure of the *MIR172b* over-expressor (MIR172bOE) construct. (C) Expression of IPS1 transcripts containing mimic transcripts with a non-cleavable *miR172* target site. (D) Expression of *MIR172b* in three independent MIR172bOE transgenic plants. Data shown are mean values from three biological replicates. Error bars ± SD. *p* values were calculated with one-way ANOVA post hoc Games-Howell test.

	Stage	Populus tremula × tremuloides	Description
Growth cessation	3	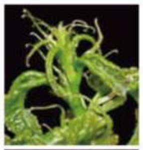	**Full active growth:** the apical shoot is in full growth, with numerous stipules and rolled-up leaves enriched at the apex; more than five embryonic leaves exhibit in the shoot.
	2.5	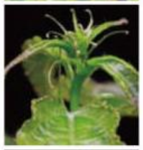	**Moderate active growth:** intermediate state between active and growth cessation state; the stipules and embryonic leaves become contact; fewer newly formed embryonic visible.
	2	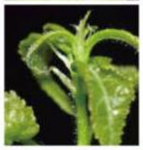	**Growth cessation:** stipules become short and no new embryonic leaves; the internodes ceased elongation; from the side view, the shoot apex tends to be horizontal.
Bud formation	1.5	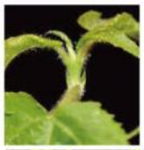	**Bud formation I:** the apex displays a inverted triangular shape. 2-3 embryonic leaves left.
	1	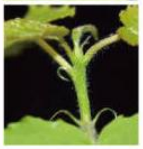	**Bud formation II:** a soft and small bud scale is visible.
Bud maturation	0.5	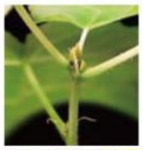	**Bud set:** the bud scale enlarges and clearly presents on the top of the shoot.
	0	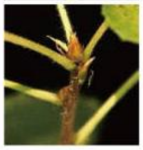	**Bud maturation:** bud scale become gradually hard with red-brown color.

Fig. S3 Scoring scheme for growth cessation and bud set in *Populus tremula x tremuloides*. A seven-stage scoring system was established to characterize complete developmental progression of growth cessation and bud set, including: full active growth (stage 3), moderate active growth (stage 2.5), growth cessation (stage 2), bud formation I (stage 1.5), bud formation II (stage 1), Bud set (stage 0.5) and Bud maturation (stage 0). This scoring system was adapted from previous studies (2) with minor modifications.

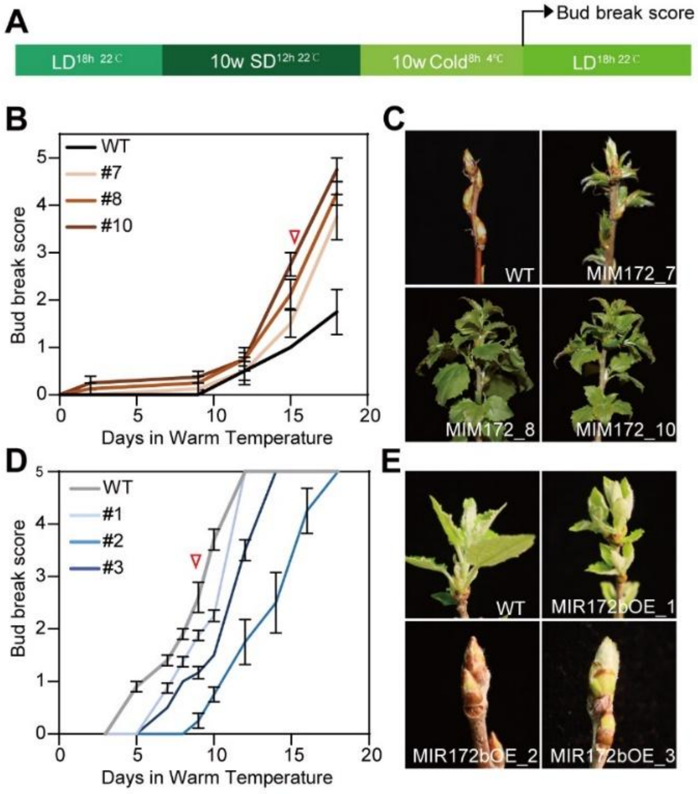

Fig. S4 Early and delayed bud break in MIM172 and MIR172bOE plants. (A) Schematic representation of the different growth conditions and the time point for bud break scoring. (B) Bud break score of wild type (WT, T89) and MIM172 plants after transfer from cold SD8h,4°C to warm LD18h, 22°C conditions. (C) Representative shoot apex morphologies of WT and MIM172 taken at 16 days after LD18h, 22°C treatment. (D) Bud break score of WT (717 background) and MIR172bOE plants. Data in (B) and (D) shown are mean values from six to eight plants of each line. Error bars ± SD. Bud break was scored as previously described (2). (E) Representative shoot apex morphologies of 717 and MIR172bOE taken at 8 days after LD18h, 22°C treatment. The corresponding score values of shoot apices in (C) and (E) are marked with red triangles in (B) and (D), respectively.

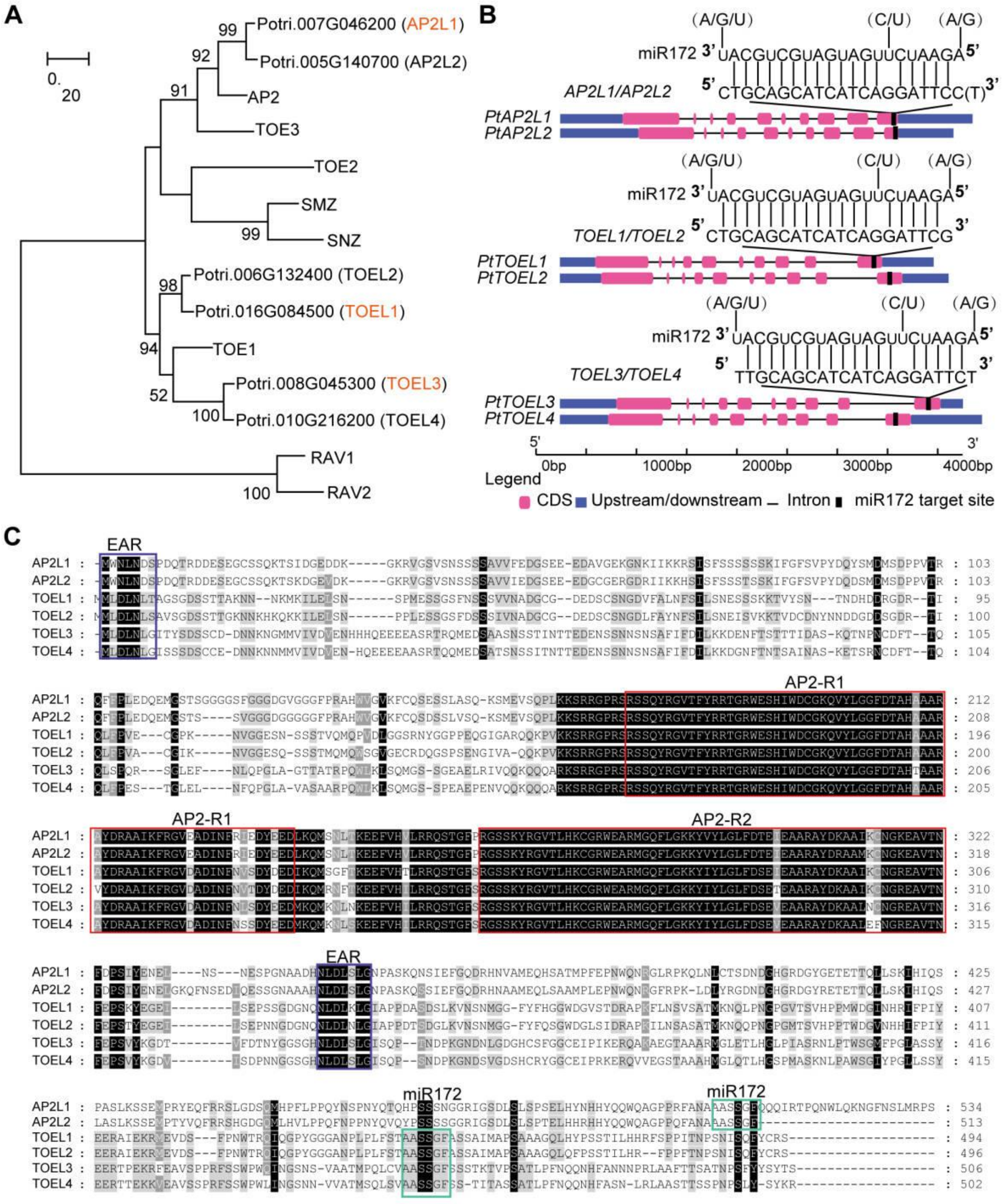

Fig. S5 Identification and characterization of AP2L/TOEL in *Populus*. (A) Phylogenetic analysis of *APETALA2-like* (*AP2-like*, euAP2 lineage) from *P. trichocarpa* and *Arabidopsis*. Phylogenetic trees were constructed with the MEGA 4.1 software using the Neighbor-Joining method. Full-length

amino acid sequences were aligned, and bootstrap analysis was performed based on 1000 replicates. Three genes that were selected for ectopic expression analysis are marked with orange color. (B) Schematic representation of gene structures of *AP2L/TOEL* and their putative *miR172* targets information. (C) Alignment of AP2L/TOEL proteins identified using ClustalX and GeneDoc. The conserved EAR motif and AP2 domains were highlighted with purple and red boxes. The amino acid coded by the miR172 target sites were also highlighted in green boxes.

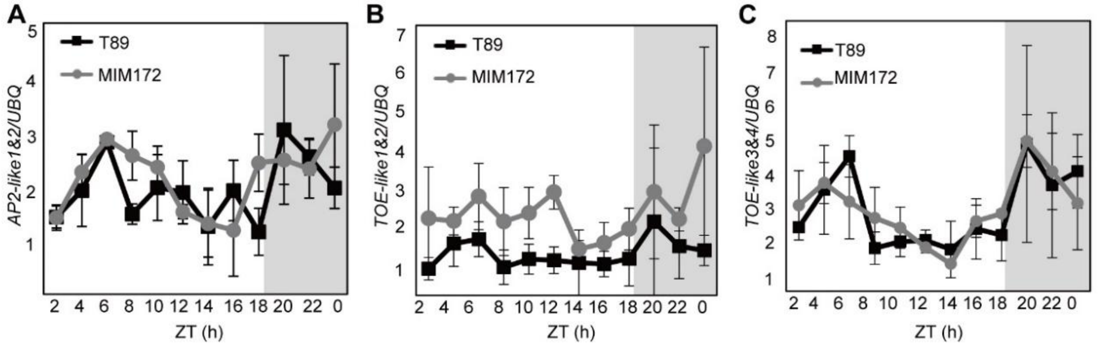

Fig. S6 Expression levels of *AP2L/TOEL1* in WT (T89) and MIM172 plants. Diurnal expression levels of *AP2L1/2* (A), *TOEL1/2* (B) and *TOEL3/4* (C) in mature leaves of WT and MIM172 plants grown under LD^{18h} conditions. Grey boxes indicate the night, white boxes indicate the day. Data shown are mean values from three biological replicates. Error bars ± SD. ZT, zeitgeber time.

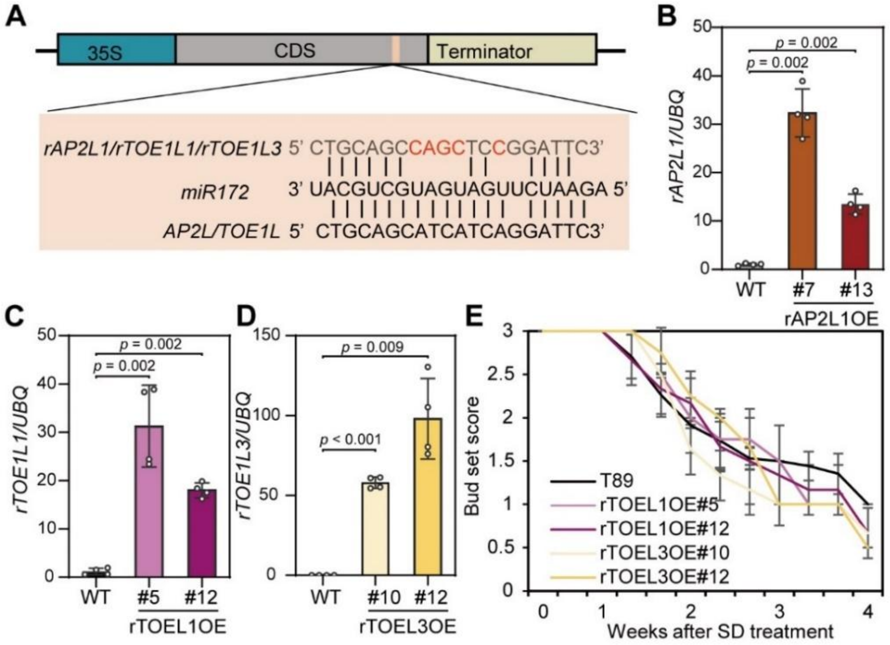

Fig. S7 Characterization of *AP2L/TOEL* over-expressors. (A) Sequences used to create the *miR172*-resistant *AP2L/TOEL* over-expression constructs. The *miR172* target sequence was substituted with a synonymous mutant sequence while still maintaining an unchanged amino acid translation sequence. (B)-(D) Expression of *rAP2L1*, *rTOEL1* and *rTOEL3* in two independent transgenic plants, respectively. Data shown are mean values from four biological replicates. *p* values were calculated with one-way ANOVA post hoc Games-Howell test. (E) Bud set score of rTOEL1OE and rTOEL3OE plants. Plants were grown under LD^{18h} growth conditions for two months, and then transferred to SD^{8h} conditions to score the growth cessation and bud set. Data shown are mean values from six plants of each line. Error bars ± SD.

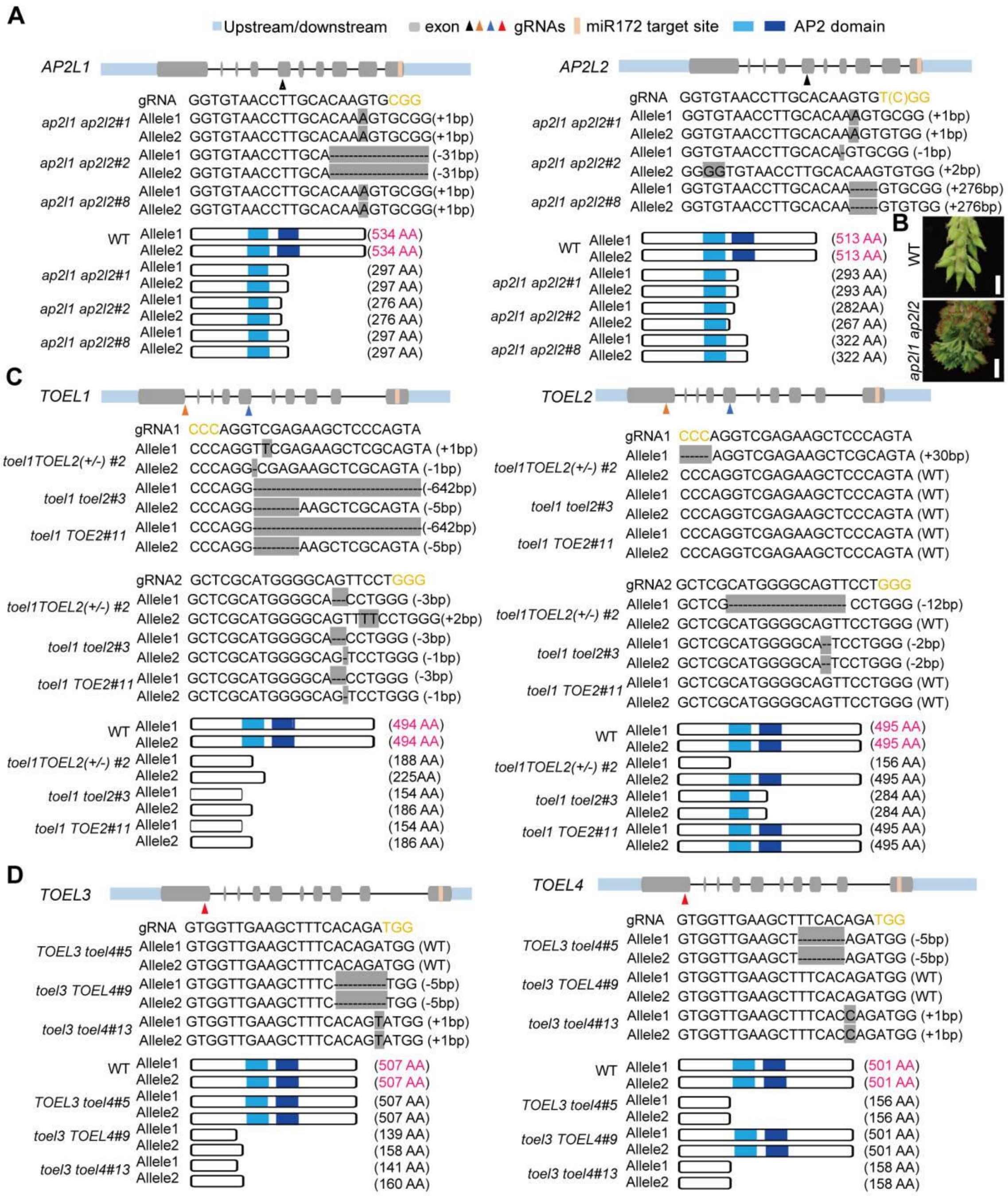

Fig. S8 Gene editing of *ap2l1 ap2l2*, *toel1 toel2* and *toel3 toel4* plants. Mutations at the sgRNA target sites of *AP2L/TOEL* genes in three independent *ap2l1 ap2l2* (A), *toel1 toel2* (C), and *toel3 toel4* (D) mutant lines, respectively. Deleted nucleotides are depicted as black dashes, and

substitutions or inserted nucleotides are represented in gray font. Yellow font indicates the protospacer-adjacent motif (PAM), and the nucleotide length of insertions and/or deletions (In/Del) is presented on the right. Further bellow showed the predicted protein length and structure of AP2L/TOEL in these mutant lines. The full-length protein of AP2L/TOEL are noted with pink color. The two DNA-binding AP2 domains (AP2-R1 and AP2-R2) are highlighted in light blue and dark blue, respectively (see alignment in Fig S5C). (B) Flower morphology of wild-type (WT) and *ap2l1 ap2l2* double mutant plants. The mutant plants exhibit severe floral developmental defects, confirming the non-functional nature of the truncated AP2L proteins.

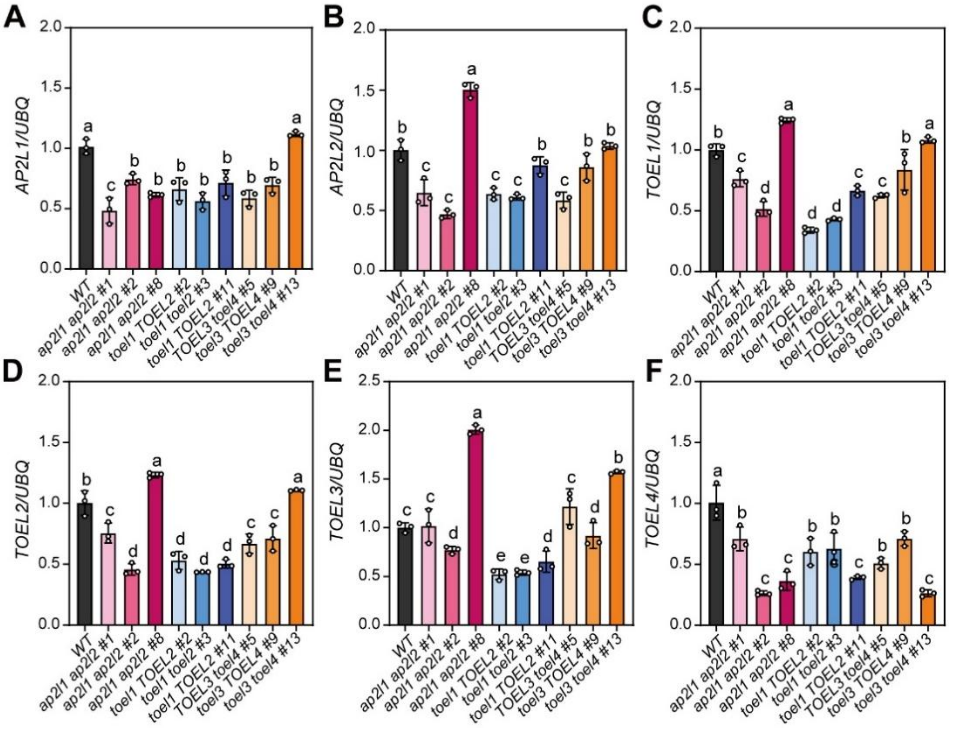

Fig. S9 Expression analysis of *AP2L* and *TOEL* genes in *ap2l* and *toel* mutants. (A-F) Relative expression levels of *AP2L1*(A), *AP2L2* (B)*, TOEL1* (C)*, TOEL2* (D)*, TOEL3* (E)*, TOEL4* (F) in *ap2l* and *toel* mutants. Data shown are mean values from three or four biological replicates. Error bars ± SD. Lowercase letters indicated statistical significance determined by one-way ANOVA post hoc Tukey's HSD test.

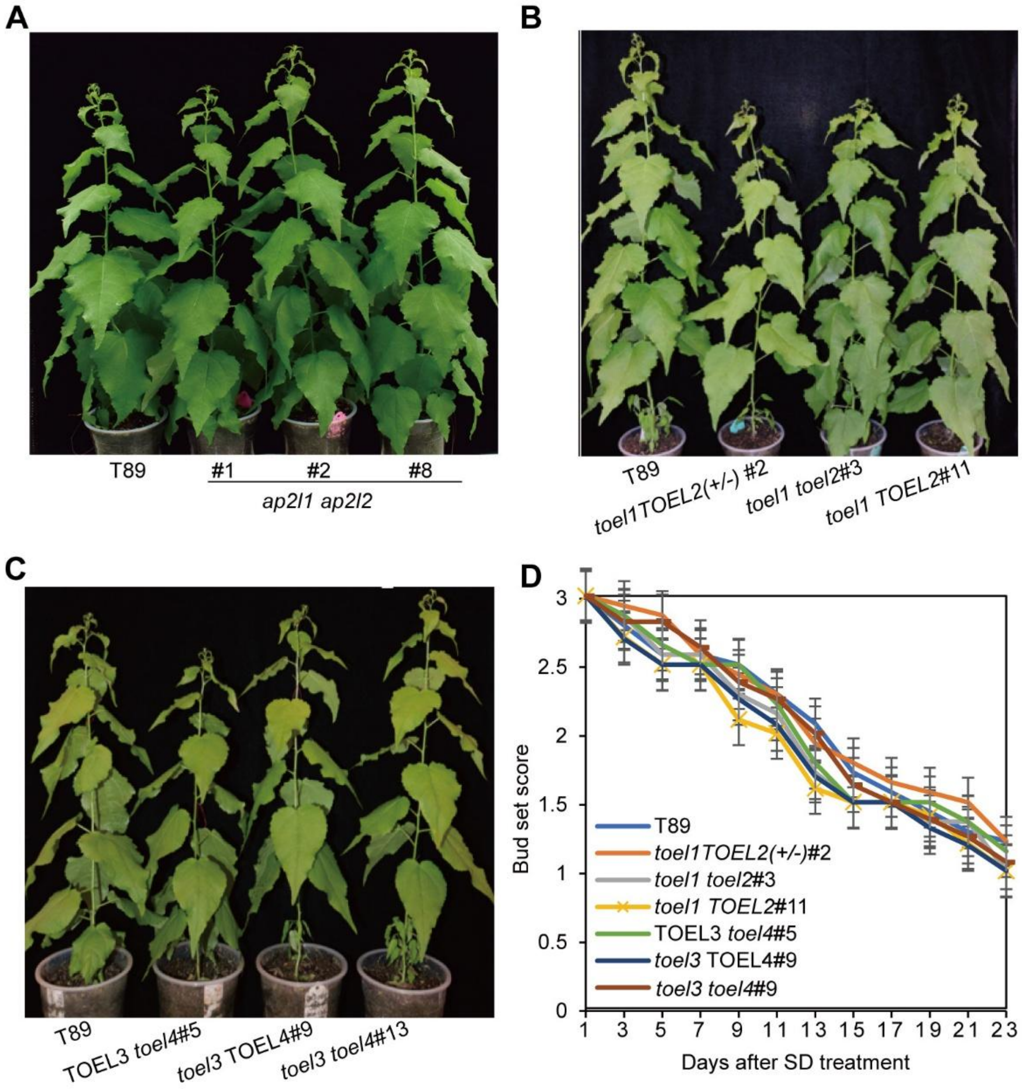

Fig. S10 Characterization of *ap2l1 ap2l2, toel1 toel2* and *toel3 toel4* transgenic plants. (A) Overview of T89 and *ap2l1 ap2l2* plants after two months of growth in LD^{18h} conditions. (B) Overview of T89 and *toel 1toel2* plants after two months of growth in LD^{18h} conditions. (C) Overview of T89 and *toel3 toel4* plants after two months of growth in LD^{18h} conditions. (D) Bud set score of *toel1 toel2* and *toel3 toel4* plants. Data shown are mean values from six plants of each line. Error bars ± SE.

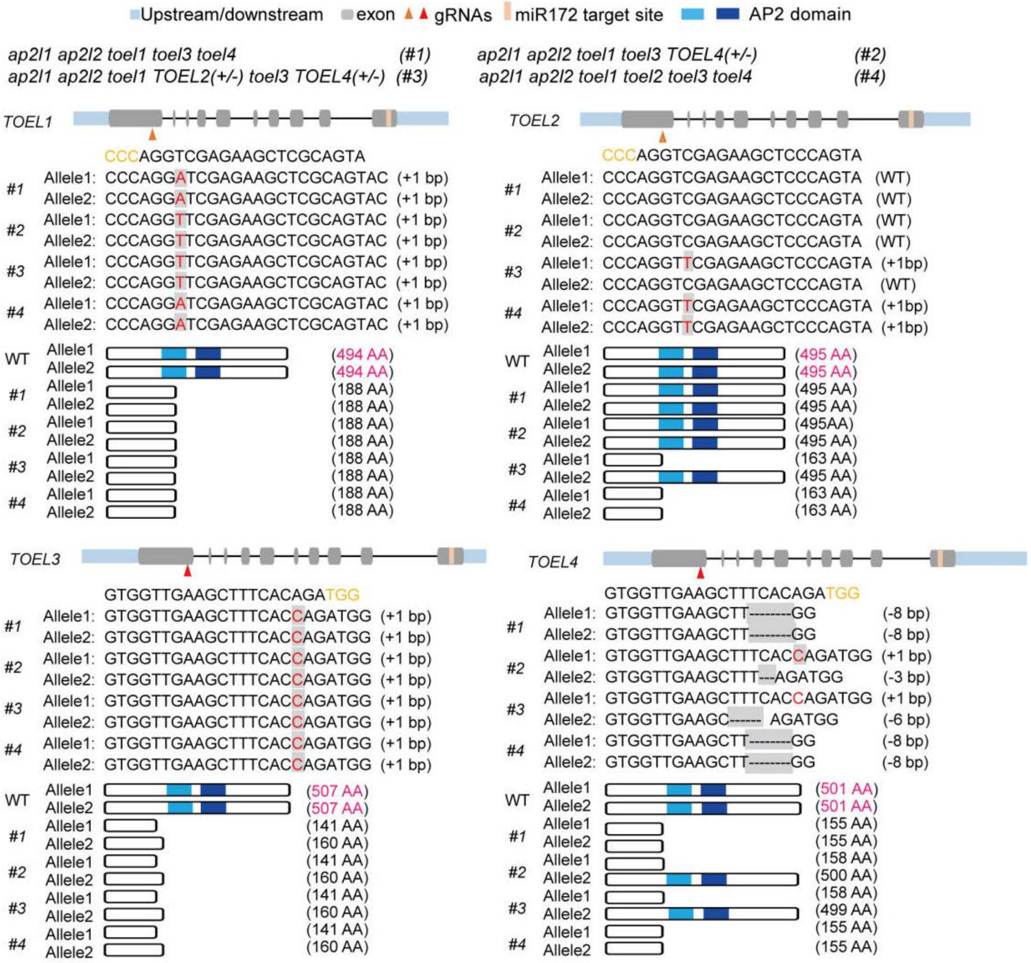

Fig. S11 Characterization of quadruple, quintuple and sextuple mutants of *AP2L/TOE1L* genes. Mutations at the sgRNA target sites for *TOEL1*, *TOEL2*, *TOEL3* and *TOEL4* in four independent *ap2l1 ap2l2 toel1 toel2 toel3 toel4* mutant lines. The quadruple, quintuple and sextuple mutants were obtained by retransforming *TOEL* knockout construct into the *ap2l1 ap2l2* #8 line. Deleted nucleotides are depicted as black dashes, and substitutions or inserted nucleotides are represented in red font. Yellow font indicates the protospacer-adjacent motif (PAM), and the nucleotide length of insertions and/or deletions (In/Del) is presented on the right. Further bellow predicted protein length and structure in these mutant lines were displayed. The full-length protein of TOEL is noted with pink color.

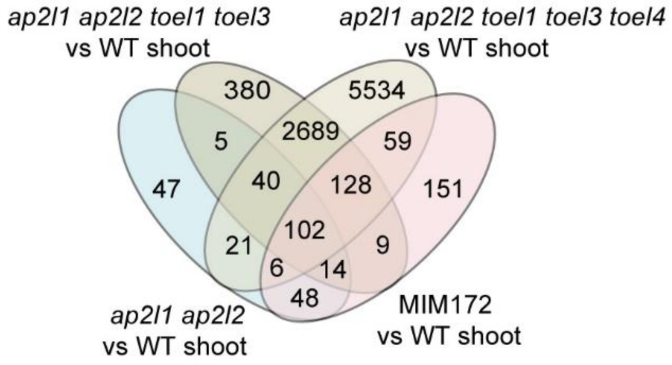

Fig. S12 Venn diagram showing the common genes among DEGs of *AP2L/TOEL* mutant lines and MIM172 plants in shoot apices from LD^{18h} conditions.

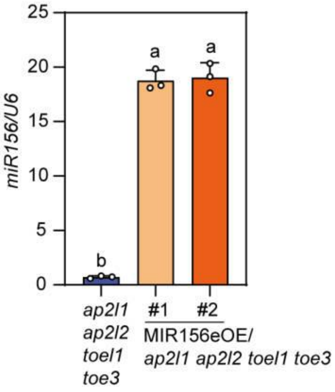

Fig. S13 Mature *miR156* expression levels in *ap2l1 ap2l2 toel1 toel3* mutants overexpressing *MIR156e*. Two independent lines were obtained, Data shown are mean values from three or four biological replicates. Error bars ± SD. Lowercase letters indicated statistical significance determined by one-way ANOVA post hoc Tukey's HSD test.

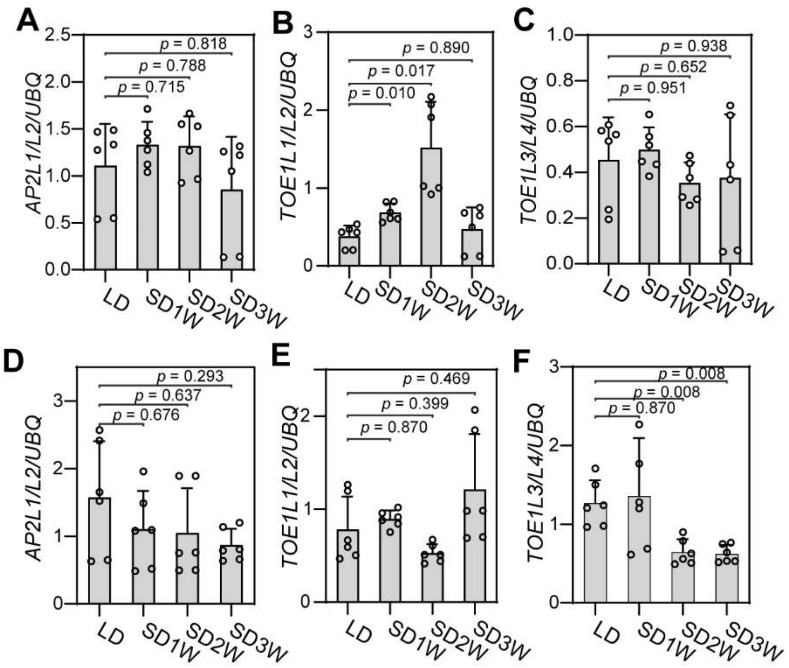

Fig. S14 *AP2L/TOEL* transcripts are not sensitive to SD photoperiod. Expression analysis of *AP2L/TOEL* genes in leaves (A-C) and shoot apices (D-F) of WT plants growing in LD^{18h} conditions (LD) and after transfer to SD^{12h} conditions (SD). Data shown are mean values from four biological replicates. Error bars ± SD. *p* values were calculated with one-way ANOVA post hoc Games-Howell test.

SI Tables

Table S1 Gene locus information of *Populus ptc-MIR172*.

Gene ID	Gene location	Strand	Length
ptc-MIR172a	Chr08:2013774..2013902	+	129
ptc-MIR172b	Chr09:1589921..1590060	-	140
ptc-MIR172c	Chr10:20940508..20940635	-	128
ptc-MIR172d	Chr01:25389766..25389947	+	182
ptc-MIR172e	Chr09:4423816..4423948	+	133
ptc-MIR172f	Chr04:22145353..22145513	+	161
ptc-MIR172g	Chr10:19351974..19352116	-	143
ptc-MIR172h	Chr08:3423592..3423734	+	143
ptc-MIR172i	Chr08:2603156..2603280	+	125

Reference genome: *Populus trichocarpa V3.0*

Dataset S1 (separate file). Prediction of miR172 target genes of *Populus trichocarpa.*

Dataset S2 (separate file). Differentially expressed genes identified in *AP2L/TOEL* mutants.

Dataset S3 (separate file). Binding sites and target genes of SPL5c identified by ChIP-seq.

Dataset S4 (separate file). Primers used in this study.

SI References

1. O. Nilsson *et al.*, Spatial pattern of cauliflower mosaic virus 35S promoter-luciferase expression in transgenic hybrid aspen trees monitored by enzymatic assay and non-destructive imaging. *Transgenic Res*, 209–220 (1992).
2. A. Rohde *et al.*, Bud set in poplar - genetic dissection of a complex trait in natural and hybrid populations. *New Phytologist* **189**, 106-121 (2011).

3. M. Todesco, I. Rubio-Somoza, J. Paz-Ares, D. Weigel, A collection of target mimics for comprehensive analysis of microRNA function in Arabidopsis thaliana. *PLoS Genet* **6**, e1001031 (2010).

4. M. Karimi, D. Inze, A. Depicker, GATEWAY((TM)) vectors for Agrobacterium-mediated plant transformation. *Trends Plant Sci* **7**, 193-195 (2002).

5. J. Mathieu, L. J. Yant, F. Murdter, F. Kuttner, M. Schmid, Repression of flowering by the miR172 target SMZ. *PLoS Biol* **7**, e1000148 (2009).

6. A. Lampropoulos *et al.*, GreenGate---a novel, versatile, and efficient cloning system for plant transgenesis. *PLoS One* **8**, e83043 (2013).

7. X. Liao *et al.*, Age-dependent seasonal growth cessation in Populus. *Proc Natl Acad Sci U S A* **120**, e2311226120 (2023).

8. O. Nilsson *et al.*, Spatial pattern of cauliflower mosaic virus 35S promoter-luciferase expression in transgenic hybrid aspen trees monitored by enzymatic assay and non-destructive imaging. *Transgenic Research* (1992).

9. Q. Liu *et al.*, Hi-TOM: a platform for high-throughput tracking of mutations induced by CRISPR/Cas systems. *Sci China Life Sci* **62**, 1-7 (2019).

10. C. N. Stewart, Jr., L. E. Via, A rapid CTAB DNA isolation technique useful for RAPD fingerprinting and other PCR applications. *Biotechniques* **14**, 748-750 (1993).

11. Q. You *et al.*, CRISPRMatch: An Automatic Calculation and Visualization Tool for High-throughput CRISPR Genome-editing Data Analysis. *Int J Biol Sci* **14**, 858-862 (2018).

12. K. Clement *et al.*, CRISPResso2 provides accurate and rapid genome editing sequence analysis. *Nat Biotechnol* **37**, 224-226 (2019).

13. J. Ding *et al.*, GIGANTEA-like genes control seasonal growth cessation in Populus. *New Phytol* **218**, 1491-1503 (2018).

14. K. Kaufmann *et al.*, Chromatin immunoprecipitation (ChIP) of plant transcription factors followed by sequencing (ChIP-SEQ) or hybridization to whole genome arrays (ChIP-CHIP). *Nat Protoc* **5**, 457-472 (2010).

15. K. M. Robinson *et al.*, An Improved Chromosome-scale Genome Assembly and Population Genetics resource for Populus tremula. *bioRxiv* 10.1101/805614, 805614 (2024).

16. Y. Rahmatallah, F. Emmert-Streib, G. Glazko, Gene set analysis approaches for RNA-seq data: performance evaluation and application guideline. *Brief Bioinform* **17**, 393-407 (2016).

17. K. J. Livak, T. D. Schmittgen, Analysis of relative gene expression data using real-time quantitative PCR and the 2(-Delta Delta C(T)) Method. *Methods* **25**, 402-408 (2001).

18. E. Varkonyi-Gasic, Stem-Loop qRT-PCR for the Detection of Plant microRNAs. *Methods Mol Biol* **1456**, 163-175 (2017).
